# Revolutionizing inflammatory bowel disease healthcare communication: a head-to-head comparison of gastroenterologist and ChatGPT responses

**DOI:** 10.1093/gastro/goaf087

**Published:** 2025-09-30

**Authors:** Zixuan He, Yilong Liu, Zhaoming Wang, Xiaoyu Zhou, Xuanming Fan, Miao He, Chengcheng Wang, Hongyu Fu, Zhijie Wang, Yu Bai

**Affiliations:** Department of Gastroenterology, Changhai Hospital, Naval Medical University, Shanghai, P. R. China; Changhai Clinical Research Unit, Changhai Hospital, Naval Medical University, Shanghai, P. R. China; Department of Gastroenterology, Shanghai Changzheng Hospital, Naval Medical University, Shanghai, P. R. China; Department of Colorectal Surgery, Changhai Hospital, Naval Medical University, Shanghai, P. R. China; Department of Gastroenterology, Changhai Hospital, Naval Medical University, Shanghai, P. R. China; Department of Gastroenterology, Changhai Hospital, Naval Medical University, Shanghai, P. R. China; Department of Pharmacy, Changhai Hospital, Naval Medical University, Shanghai, P. R. China; Department of Clinical Nutrition, Changhai Hospital, Naval Medical University, Shanghai, P. R. China; National Clinical Research Center for Digestive Diseases (Shanghai), Shanghai, P. R. China; Department of Gastroenterology, Affiliated Hangzhou First People’s Hospital, Westlake University School of Medicine, Hangzhou, Zhejiang, P. R. China; Department of Gastroenterology, Changhai Hospital, Naval Medical University, Shanghai, P. R. China; Changhai Clinical Research Unit, Changhai Hospital, Naval Medical University, Shanghai, P. R. China

**Keywords:** inflammatory bowel disease, large language models, GPT-4 Omni, patient education

## Abstract

**Background:**

Artificial intelligence-driven large language models demonstrate immense potential in the medical field. It remains unclear whether ChatGPT has the ability to provide appropriate recommendations for patients with inflammatory bowel disease (IBD) that are comparable to those of gastroenterologists. This study quantitatively assessed the performance of ChatGPT’s generated IBD-related recommendations from the distinct perspectives of gastroenterologists and patients.

**Methods:**

Healthcare questions regarding IBD were solicited from IBD patients and specialized physicians. Those questions were then presented to GPT-4 Omni and three independent senior gastroenterologists for responses. These responses were subsequently evaluated by a blinded panel of five board-certified gastroenterologists using a five-point Likert scale, assessing accuracy, completeness, and readability. Furthermore, 10 IBD patients as blinded assessors performed assessments of both ChatGPT’s and gastroenterologists’ responses.

**Results:**

Thirty high-frequency questions were selected, encompassing basic knowledge, treatment, and management domains. ChatGPT demonstrated high reproducibility in responding to these questions. Regarding accuracy and readability, ChatGPT’s performance was comparable to that of gastroenterologists. For completeness of responses, ChatGPT outperformed gastroenterologists (4.42 ± 0.67 vs 4.19 ± 0.65; *P *= 0.012). Overall, IBD patients were satisfied with both ChatGPT’s and gastroenterologists’ responses but, for treatment-related questions, patients rated gastroenterologists higher than ChatGPT (4.54 ± 0.32 vs 4.21 ± 0.38; *P *= 0.040).

**Conclusions:**

ChatGPT has the potential to provide stable, accurate, comprehensive, and comprehensible healthcare-related information for IBD patients. Further validation of the reliability and practicality of large language models in real-world clinical settings is crucial.

## Introduction

Inflammatory bowel disease (IBD) encompasses a spectrum of chronic gastrointestinal disorders characterized by persistent inflammation. A robust comprehension of the disease is pivotal for improving clinical outcomes, as it enhances treatment adherence and facilitates shared decision-making between patients and clinicians [1]. Nevertheless, research employing the Crohn’s and Colitis Knowledge Score indicates substantial gaps in disease-related knowledge among patients with IBD, which may propel their active information seeking through various channels, including online platforms and physician consultations [2].

The advent of artificial intelligence (AI)-driven large language models (LLMs), particularly ChatGPT, has revolutionized natural language processing with far-reaching implications for healthcare [3]. ChatGPT’s capability in understanding and processing medical information hints at its potential across various healthcare domains, including medical education and clinical support [4–6]. Specifically, ChatGPT shows promise in disease prevention, diagnosis, treatment, monitoring, and patient care. Studies comparing ChatGPT’s performance against physicians in addressing patient queries and conducting medical assessments have commenced [7–9].

Studies in gastroenterology have explored ChatGPT’s accuracy in answering medical questions [10]. Pugliese *et al.* [11] reported ChatGPT’s highly comprehensible responses for questions about nonalcoholic fatty liver disease, while Henson *et al.* [12] found its responses appropriate for most gastroesophageal reflux disease-related queries. Yeo *et al.* [9] noted ChatGPT’s ability to address cirrhosis and liver cancer questions, albeit with room for improvement in completeness. Similarly, Gravina *et al.* [13] compared ChatGPT’s IBD-related responses with clinical guidelines and found scientific reliability but a lack of updated data; crucially, the study lacked quantitative assessment. Sciberras *et al.* [14] demonstrated the accuracy of ChatGPT in answering questions related to patients with IBD through physician evaluations, with results showing that ChatGPT achieved an accuracy rate of 84.2% across various clinical scenarios. A similar outcome was observed in the study by Ghersin *et al.*, in which ChatGPT attained an accuracy rate of 87.8% [15]. However, none of the above studies considered the perspective of IBD patients. Therefore, this study aims to quantitatively assess the performance of ChatGPT’s responses to IBD-related fundamental knowledge, treatment, and daily management recommendations, comparing them with those of gastroenterologists from both professional and patients’ perspectives.

## Methods

### Data source

The study was conducted in May 2024, drawing participants from the outpatient and inpatient departments of gastroenterology at Changhai Hospital. Patients were invited by their treating physicians or via posters displayed in clinic waiting areas. To ensure the comprehensive coverage of IBD-related concerns, we invited 78 patients (40 with ulcerative colitis, 38 with Crohn’s disease) and 10 specialists (gastroenterologists, surgeons, dietitians, and pharmacists) to gather their most pressing or frequently asked questions. Similar questions were combined and ambiguous ones were excluded. Some modifications were made to the wording and grammar of certain questions to ensure precise articulation. This initiative was conducted from 1 to 27 May 2024.

### Study design

**Figure 1.**
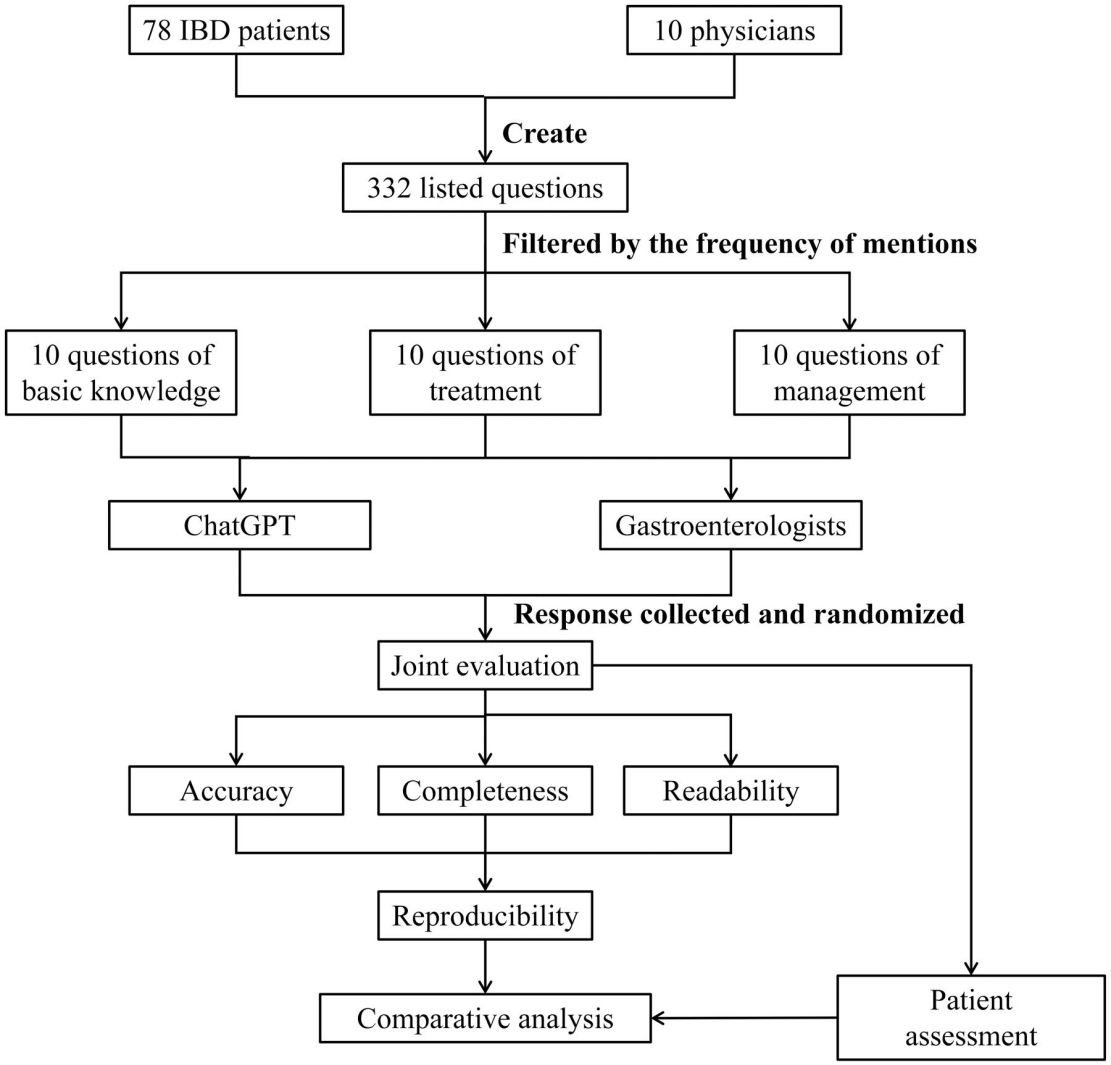
Flowchart of the study.

Firstly, the highest-attention questions were sequentially fed into GPT-4 Omni (GPT-4o; May 28–30 version). Before each question was entered, the statement “I am a patient with IBD” was posed on the online AI interface to ensure that subsequent responses would be based on this premise. Each question was entered as a separate and independent prompt by using the “New Chat” feature in Chinese. Each question was asked once a day for 3 consecutive days. Secondly, three senior gastroenterologists were invited to review and provide their answers to these questions. All responses from ChatGPT and the gastroenterologists were systematically documented for subsequent analysis. This study was determined to be exempt from review by the institutional review board of Changhai Hospital and all patients reviewed and signed the informed consent. The study adhered to the Strengthening the Reporting of Observational Studies in Epidemiology reporting guideline. The study flowchart is presented in Figure 1.

### Response grading

The human-written and AI-generated responses to each question were subsequently reviewed and graded by a masked panel of five board-certified gastroenterologists following a joint discussion. The entire research process, encompassing questions formulation, responses provision, and the blinded grading, was managed by three distinct and independent teams of gastroenterologists, each with >10 years of clinical experience in IBD.

The reviewers were asked to evaluate each response for accuracy, completeness, and patient readability based on the IBD guidelines and their clinical experience by using a five-point Likert scale for quantitative scoring [16–20]. For accuracy, a score of 1 represents a completely incorrect response and 5 is entirely correct. For completeness, a score of 1 stood for an answer that only addressed some aspects of the question with significant parts missing or incomplete, while 5 denoted a comprehensive response that covered all aspects of the question and offered additional information or context beyond expectations. For readability, a score of 1 means that the response is overly complex, filled with jargon, or poorly organized, while 5 means that the response was clear, concise, and accessible to a wide range of readers, regardless of their background or familiarity with the topic. To ensure the integrity of the blind, we uniformly processed all collected responses, standardizing their format and concealing any distinctive features of chatbots or human experts. This initiative was conducted from 1 June to 3 July 2024.

### Patients’ evaluation

Considering that these responses are tailored to the IBD patient population, 10 patients with IBD were invited to evaluate the responses independently, without knowing whether they were generated by ChatGPT or by gastroenterologists. Patients first read the questions and their corresponding answers, and then quantitatively scored the quality of the responses by using a five-point Likert scale. Specifically, a score of 1 indicated no understanding at all and was not helpful, whereas a score of 5 indicated easy understanding and high helpfulness. Finally, they could comment on any of the responses, including their strengths and weaknesses. This initiative was conducted from 15 to 30 July 2024.

### Statistical analysis

The Kolmogorov–Smirnov test was employed to assess the normality of the continuous variable. As the data were not normally distributed, nonparametric statistical tests were utilized for analysis. Consequently, the Mann–Whitney *U* test was used to compare the distributions of both the experts’ scores and the patients’ scores between responses generated by ChatGPT and those by gastroenterologists. Subsequently, the reproducibility of the response from ChatGPT was assessed to compute the intraclass correlation coefficient. The intraclass correlation coefficient ranged from 0 to 1, with the following established benchmarks for consistency: <0.2 (poor), 0.2–0.4 (general), 0.4–0.6 (moderate), 0.6–0.8 (good), and 0.8–1.0 (excellent). A *P* value of <0.05 was considered statistically significant. Statistical analyses were conducted by using SPSS software (Version 22.0; SPSS Inc., Chicago, IL, USA).

## Results

### Enumeration of IBD-related queries

After duplicates and unclear questions were excluded, a total of 332 IBD-related questions were collected from 78 IBD patients and 10 specialists. Based on the frequency of mentions, we selected the top 10 questions of greatest interest for each category (basic knowledge, treatment, and management), yielding total of 30 questions for subsequent evaluation (Table 1). Overall, each of these 30 questions was mentioned with a frequency of >40%, underscoring their significance and representativeness as the most desired and common information among IBD patients.

**Table 1.** Top 10 questions in IBD: basic knowledge, treatment, and daily management

Question	Frequency of mentions
Basics	
1. Why do I have IBD?	54
2. What is the difference between IBD and infectious enteritis?	36
3. Why do I need to undergo so many tests to confirm the diagnosis of IBD?	48
4. What is the difference between ulcerative colitis and Crohn’s disease?	38
5. What are the clinical symptoms of IBD?	46
6. Why do I often feel tired?	51
7. What are the extra-intestinal manifestations of IBD?	37
8. Will I turn into colorectal cancer?	42
9. Will IBD have an impact on my life expectancy?	53
10. Will IBD be passed on to my children?	36
Treatment	
1. Can corticosteroids be used for a long time?	44
2. Can immunosuppressants be used for a long time?	41
3. When can I be treated with biologics?	37
4. How do I choose the right biologic for me?	46
5. When do I need to undergo surgical treatment?	39
6. What is upadacitinib?	36
7. When do I need enteral nutrition?	42
8. Is fecal transplantation useful?	40
9. Can IBD be cured?	56
10. What is the ultimate therapeutic goal for IBD?	40
Daily management	
1. What is the best diet for IBD?	56
2. What kind of foods should I avoid?	51
3. How should I determine if the current treatment is working for me?	45
4. How often do I need to have a colonoscopy?	50
5. What is the use of fecal calprotectin testing?	43
6. How can I prevent intestinal infections?	45
7. How can I relieve my anxiety and pessimism?	44
8. Will I need lifelong treatment?	42
9. What can I do to prevent a relapse?	42
10. How can I tell if I am in the acute or remission phase?	41

### Response grading

**Figure 2.**
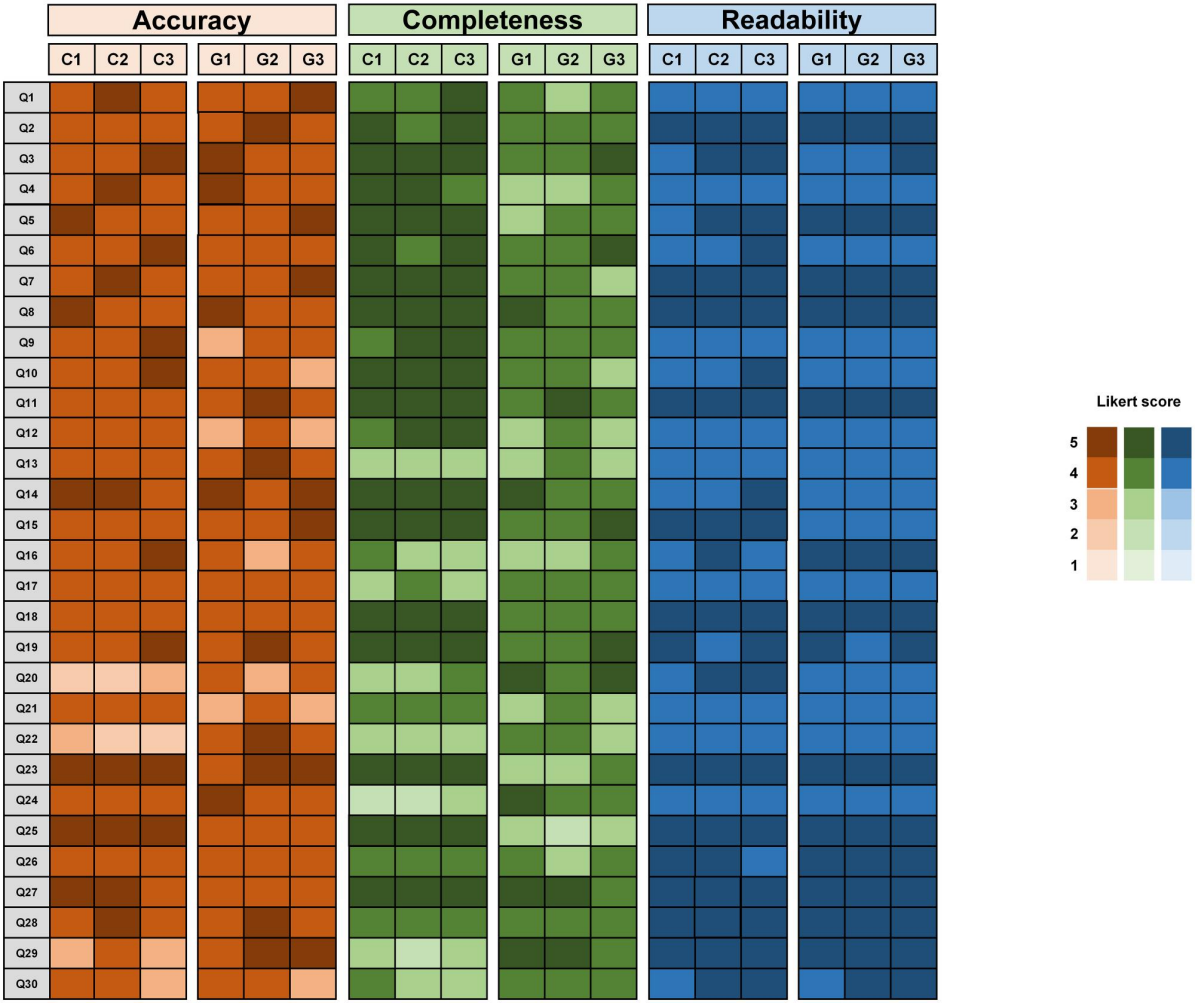
Details of the response assessment using a five-point Likert scale from gastroenterologists and ChatGPT in terms of accuracy, completeness, and readability.

ChatGPT demonstrated high levels of reproducibility in responding to 30 questions from 3 domains. The intraclass correlation coefficients for accuracy, completeness, and readability of the responses were 0.89, 0.90, and 0.86, respectively. The details of response assessments from gastroenterologists and ChatGPT are shown in Figure 2.

**Figure 3.**
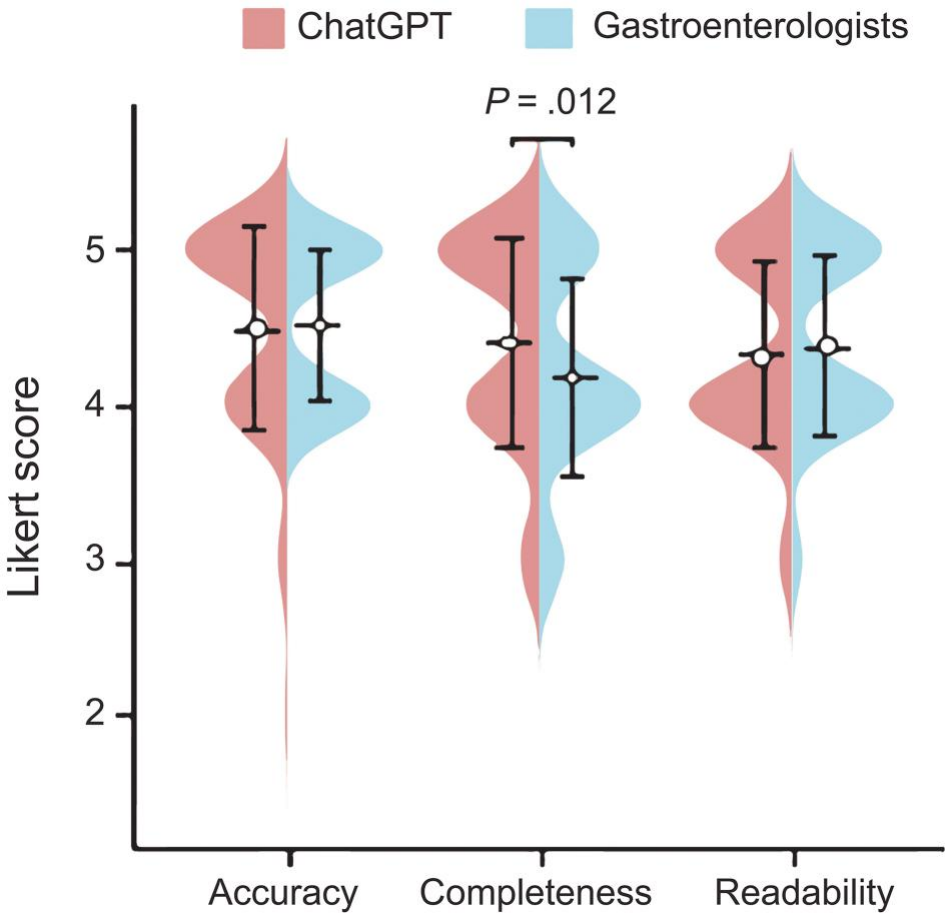
Accuracy, completeness, and readability of the responses of ChatGPT and gastroenterologists.

In terms of accuracy, ChatGPT (4.50 ± 0.65) demonstrated a comparable level to that of gastroenterologists (4.53 ± 0.50; *P *= 0.833) (Table 2 and Figure 3). Furthermore, this result remained consistent across the three distinct domains of basic knowledge, treatment, and management. However, ChatGPT provided inaccurate answers to certain questions. Specifically, two questions (“How often do I need to have a colonoscopy?” and “What kind of foods should I avoid?”) were evaluated as inappropriate (average Likert score of ≤3).

**Table 2.** Comparison of the Likert scores for accuracy, completeness, and readability of responses from ChatGPT and gastroenterologists

Item	ChatGPT		Gastroenterologists		*P* value
	Mean ± SD	Median (IQR)	Mean ± SD	Median (IQR)	
Overall accuracy	4.50 ± 0.65	5 (4, 5)	4.53 ± 0.50	5 (4, 5)	0.833
Basics	4.73 ± 0.45	5 (4, 5)	4.73 ± 0.45	5 (4, 5)	>0.999
Treatment	4.40 ± 0.50	4 (4, 5)	4.43 ± 0.50	4 (4, 5)	>0.999
Management	4.37 ± 0.89	5 (4, 5)	4.43 ± 0.50	4 (4, 5)	0.736
Overall completeness	4.42 ± 0.67	5 (4, 5)	4.19 ± 0.65	4 (4, 5)	0.012*
Basics	4.70 ± 0.47	5 (4, 5)	4.23 ± 0.57	4 (4, 5)	0.002*
Treatment	4.10 ± 0.99	4 (4, 5)	4.23 ± 0.68	4 (4, 5)	0.828
Management	4.30 ± 0.79	4.5 (4, 5)	4.10 ± 0.71	4 (4, 5)	0.261
Overall readability	4.33 ± 0.60	4 (4, 5)	4.39 ± 0.59	4 (4, 5)	0.554
Basics	4.13 ± 0.97	4 (4, 5)	4.27 ± 0.58	4 (4, 5)	0.894
Treatment	4.33 ± 0.61	4 (4, 5)	4.30 ± 0.60	4 (4, 5)	0.894
Management	4.40 ± 0.62	4 (4, 5)	4.60 ± 0.56	5 (4, 5)	0.235

SD = standard deviation, IQR = interquartile range.

* *P* < 0.05.

Regarding completeness of responses, ChatGPT demonstrated an advantage over gastroenterologists (4.42 ± 0.67 vs 4.19 ± 0.65, respectively; *P *= 0.012). The subgroup analysis revealed that ChatGPT’s performance in answering treatment and management questions did not differ significantly from those of gastroenterologists. However, ChatGPT significantly outperformed gastroenterologists when answering questions on the basics of IBD (4.70 ± 0.47 vs 4.23 ± 0.57, respectively; *P *= 0.002). Nevertheless, there was a lack of relevant details and more precise guidance from ChatGPT in addressing queries concerning upadacitinib, dietary restrictions, as well as the follow-up interval of colonoscopy (Table 2).

In terms of readability, ChatGPT provided answers to the majority of questions in easily understandable language (4.33 ± 0.60), avoiding excessive use of complex medical terminology (Table 2). Its performance was comparable to those of gastroenterologists (4.39 ± 0.59; *P *= 0.554). This finding also held true across the three subgroups of basic knowledge, treatment, and management. Detailed responses for all questions are available in Supplementary Table 1.

### Patient-centered evaluation

Table 3 presents the results of the evaluations by IBD patients. Overall, patients were satisfied with the responses from both ChatGPT and the gastroenterologists, with mean Likert scores of >4. No significant difference was observed in overall scores between the two groups. Subgroup analyses were performed to assess the comparability of ChatGPT’s responses to those of gastroenterologists across all domains. The results showed that ChatGPT and the gastroenterologists performed comparably when addressing questions on basic knowledge and management. Interestingly, in terms of treatment, patients rated gastroenterologists’ responses higher than ChatGPT’s (4.54 ± 0.32 vs 4.21 ± 0.38, respectively; *P *= 0.040). Patient feedback suggested that this preference was attributed to the brevity and focus of the gastroenterologists’ responses.

**Table 3.** Comparison of the responses from ChatGPT and gastroenterologists, as evaluated by IBD patients

Item	ChatGPT		Gastroenterologists		*P* value
	Mean ± SD	Median (IQR)	Mean±SD	Median (IQR)	
Overall	4.37 ± 0.35	4 (4, 5)	4.43 ± 0.31	4 (4, 5)	0.440
Basics	4.61 ± 0.23	5 (4, 5)	4.35 ± 0.38	4 (4, 5)	0.124
Treatment	4.21 ± 0.38	4 (4, 5)	4.54 ± 0.32	5 (4, 5)	0.040^*^
Daily management	4.28 ± 0.31	5 (4, 5)	4.39 ± 0.22	4 (4, 4)	0.343

SD = standard deviation, IQR = interquartile range.

* *P* < 0.05.

## Discussion

This study provides a detailed evaluation of ChatGPT’s ability to address the most concerning issues of IBD patients. Utilizing a rigorous research design with appropriate blinding and randomization, we compared ChatGPT’s performance against those of gastroenterologists across multiple domains. The findings suggested that ChatGPT possesses the potential to offer appropriate information on basic knowledge, treatment, and daily management recommendations for IBD patients, demonstrating good reproducibility. In terms of accuracy and readability, ChatGPT performs comparably to gastroenterologists. Interestingly, ChatGPT generally outperformed gastroenterologists in response completeness, especially regarding fundamental knowledge. For example, when asked “What is the difference between ulcerative colitis and Crohn’s disease?”, ChatGPT thoroughly summarized the distinctions, including clinical symptoms, complications, endoscopic appearances, and pathological features, while gastroenterologists may emphasize key points rather than detailing every aspect. This difference might arise because gastroenterologists, in their daily clinical practice, frequently prioritize treatment approaches and daily management, often highlighting main points when explaining fundamental knowledge in fast-paced medical settings. A recent study has reported comparable accuracy between ChatGPT and IBD experts for IBD-related queries [15]. Another study, based on European Crohn’s and Colitis Organisation guidelines, concluded that ChatGPT can provide accurate and comprehensive responses to practical questions from IBD patients [14]. In our patients’ evaluation component, respondents generally rated ChatGPT and gastroenterologists similarly in overall response quality. However, for treatment-related questions, patients rated gastroenterologists’ responses to be more understandable, actionable, and helpful than ChatGPT’s. This inconsistency with physician evaluations likely stems from ChatGPT’s occasional use of ambiguous words in responses such as “possibly” or “approximately”, which may confuse patients, whereas physicians’ more precise language makes patients convinced. Supporting our findings, previous studies have found that ChatGPT demonstrates adequate accuracy when responding to patient inquiries about gastrointestinal diseases [12,13, 21]. It is deemed acceptable and exhibits high clarity and user satisfaction [6, 22]. Furthermore, the reliability of AI-generated medical information extends beyond gastroenterology, with studies confirming its utility in other domains such as ophthalmology, cardiovascular disease prevention, and clinical pharmacy [23–25].

Despite its potential, ChatGPT exhibits notable limitations. LLMs are limited by their training data, which may preclude an in-depth understanding of medical concepts, thus rendering their accuracy reliant on the quality of that data. To our knowledge, the latest LLMs such as GPT-4o have not undergone specialized medical training, which could pose a risk of providing incorrect clinical information or making erroneous clinical decisions. Our study identified instances of such inaccuracies for certain questions. For example, when asked about the timing of endoscopic assessments for IBD patients, ChatGPT erroneously recommended annual assessments for those in the active phase, deviating from the STRIDE initiative from the International Organization for the Study of Inflammatory Bowel Diseases (IOIBD) [20], which recommends endoscopic assessments at a minimum of 3 months during active ulcerative colitis and 6- to 9-month intervals during active Crohn’s disease. Similarly, ChatGPT’s recommendations for colonoscopy screening and surveillance for colorectal cancer in IBD patients were generally imprecise, suggesting surveillance colonoscopy every 1–2 years starting 8 years after the onset of initial symptoms. Notably, it failed to provide tailored surveillance strategies based on the risk stratification of colorectal cancer [26], which is crucial for optimizing care and reducing the risk of colorectal cancer in high-risk IBD patients. Furthermore, ChatGPT’s advice on foods to avoid for IBD patients, specifically its negation of all high-fiber foods and dairy products, suggests that these might exacerbate symptoms. However, these suggestions are overly generalized and unsubstantiated. According to the latest dietary guidance from the IOIBD and American Gastroenterological Association clinical practice update on diet and nutritional therapies, a Mediterranean diet rich in various fresh fruits and vegetables should be recommended for all patients with IBD unless contraindicated [19, 27]. These weaknesses may stem from outdated training datasets or the inability to assess and grade the reliability of data sources, such as prioritizing the latest guidelines and literature over medical media blogs [28]. Furthermore, the lack of sources for ChatGPT’s information undermines user trust, as it fails to provide a transparent foundation for its recommendations. Although ChatGPT proves reliable for general and common queries, its inability to offer personalized information tailored to individual patients’ circumstances remains a limitation [29].

Unlike previous studies, which evaluated ChatGPT’s performance by answering preset medical questions pertaining to digestive diseases [13, 14], our study introduces several novel aspects. Firstly, both IBD patients and physicians collaborated in screening questions, selecting those most frequently asked during the illness trajectory and categorizing them into distinct domains. Critically, each question included in our study was proposed by ≥40% of participants. Furthermore, in assessing ChatGPT’s responses versus those of experienced gastroenterologists, we randomized responses from different sources and blinded the raters to avoid potential subjective bias. To our knowledge, this is the first study in the field of digestive diseases to have implemented such randomization and blinding in comparing ChatGPT’s and healthcare providers’ responses. Moreover, considering that the main purpose of this study is to assess whether ChatGPT can provide reliable medical assistance to patients, we evaluated ChatGPT’s responses from the perspective of patients. Unlike evaluations by healthcare professionals, patient-centered assessments can determine whether ChatGPT is suitable for real medical scenarios—namely, whether the answers are easily understood and effectively address the issues. Lastly, the study is the first to have conducted interactions with ChatGPT on IBD in Chinese. With the advance of globalization, multilingual interaction has become an important requirement. ChatGPT can understand the cultural background of Chinese users and output answers with corresponding language habits and modes of thinking, thereby demonstrating its strong ability in cultural integration and multi-language interaction.

There are some limitations of this study. First, despite selecting and evaluating IBD-related questions that are of most interest to patients, the field of IBD is multifaceted and rapidly updating, making it challenging to comprehensively cover all patient concerns. Second, potential bias in the outcomes from subjective assessments is unavoidable, in both physician and patient assessments. Third, we did not conduct a cross-comparison of multiple LLMs because, although GPT4o offers enhanced functionalities, it is a paid service, which might limit its accessibility for patient applications. Furthermore, our current evaluation did not quantify the potential economic benefits or cost-saving implications of AI implementation in clinical practice; future research should incorporate detailed cost-effectiveness analyses that compare traditional care models with AI-assisted approaches, including metrics such as consultation time reduction, clinician labor redistribution, and long-term patient self-management outcomes. Considering the limitations of this study, future research should incorporate larger sample sizes and adopt more objective evaluation metrics to further assess the performance of LLMs in real-world clinical settings.

## Conclusions

ChatGPT provides accurate, readable, and comprehensive medical information for IBD patients, matching gastroenterologists in accuracy and readability while offering more complete responses, especially for basic knowledge. However, limitations such as occasional inaccuracies and lack of personalization remain. Despite these issues, ChatGPT shows potential as a supplementary patient education tool. Future work should refine AI models with updated guidelines, improve transparency, and enhance personalization for real-world clinical use.

## Supplementary Material

goaf087_Supplementary_Data

## Data Availability

The datasets generated and analysed during the study are available by contacting the corresponding author at baiyu1998@hotmail.com.
