# Supplementary material for: Revolutionizing inflammatory bowel disease healthcare communication: a head-to-head comparison of gastroenterologist and ChatGPT responses
Source: Gastroenterol Rep (Oxf). 2025 Sep 30;13:goaf087. doi: 10.1093/gastro/goaf087 (PMC12479395; doi:10.1093/gastro/goaf087)
Supplement: goaf087_Supplementary_Data [file goaf087_supplementary_data.docx]

**Supplementary table 1. Detailed responses to all questions.**

|  | Responses from the GPT 4o | | | Responses from the gastroenterologists | | |
| --- | --- | --- | --- | --- | --- | --- |
|  | Response 1 | Response 2 | Response 3 | Response 1 | Response 2 | Response 3 |
| **Basics** | | | | | | |
| 1. Why do I have IBD? | Although the specific causes of IBD are not fully understood, the following factors may contribute to the disease: 1 Genetic factors: If someone in your family has IBD, the risk of developing the disease is higher. This suggests that genes may play a role in it. Immune system problems: The immune system may overreact to normal bacteria or food in the gut, causing inflammation. Environmental factors: Certain environmental factors, such as infections, diet, stress, and smoking, may increase the risk of developing the disease. For example, in some countries and regions, the incidence rate of IBD is higher. Lifestyle: Although lifestyle choices are not the direct cause, they can affect the severity of the condition. For example, stress, smoking and unhealthy diet may aggravate the condition. | Although the specific cause of IBD is not fully understood, there are several factors that may contribute to the occurrence of this disease: Genetic factors: Having a family member with IBD increases your risk of developing the disease, suggesting that genetics may play a role. Abnormal immune system: The immune system may overreact to normal bacteria or food in the gut, leading to intestinal inflammation. Environmental factors: Certain environmental conditions, such as infections, dietary habits, stress, and smoking, may increase the risk of developing the disease. For example, in some areas, the incidence rate of IBD is high. Lifestyle: While lifestyle choices are not the direct cause, they may influence the severity of the condition. For example, excessive stress, smoking, and unhealthy diet may aggravate the condition. | The exact cause of IBD is not fully understood, but the following factors may be related to its onset: 1 Genetic factors: If someone in the family has IBD, the risk of disease increases, suggesting that genes may play a role in the onset of the disease. 2. Immune system problems: The immune system may have an abnormal reaction to normal bacteria or food in the intestines, causing inflammation in the intestines. Environmental factors: Certain environmental factors, such as infections, eating habits, stress, and smoking, may increase the risk of developing the disease. For example, in some countries and regions, the incidence rate of IBD is high. Lifestyle: Although lifestyle is not a direct cause of the disease, it can affect the severity of the condition. For example, excessive stress, smoking, and unhealthy diet may exacerbate the condition. | The occurrence of IBD involves multiple potential factors, and although the specific causes are not fully understood, the following factors may be related to it. Firstly, genetic factors play a role in the onset of IBD, as the disease tends to cluster in families, and individuals with a history of enteritis in their family are more susceptible to it. Secondly, immune factors are also believed to be related to IBD, as immune system dysfunction or autoimmune diseases may trigger intestinal inflammation. Bacterial infection is another factor, and poor diet may interfere with the balance of the gastrointestinal tract, leading to bacterial infection and gastrointestinal diseases. In addition, bad habits such as frequent consumption of carbonated drinks, spicy and stimulating foods, and smoking may also lead to IBD. | IBD is a disease caused by multiple factors interacting with each other, and there may be different combinations of causes among different patients. IBD has a genetic predisposition, and the incidence rate of first-degree relatives of patients is significantly higher than that of the general population. Abnormal intestinal mucosal immune system, improper diet, smoking, poor sanitation, mental stimulation, excessive fatigue, and eating disorders are also associated with the occurrence of IBD. | IBD is a chronic inflammatory bowel disease of unknown etiology, including ulcerative colitis and Crohn's disease. The pathogenesis of this condition is not fully understood, but it may be related to the interaction of various factors such as environment, genetics, infection, immunity, and lifestyle. For example, long-term exposure to high-pressure work and living environments, relevant family history, bacterial infection, intestinal immune system disorder, long-term consumption of unhealthy foods such as fast food and take-out food. |
| 2. What is the difference between IBD and infectious enteritis? | Although both IBD and infectious enteritis affect the intestine and cause similar symptoms, they have different causes and characteristics. IBD is a chronic inflammation caused by an abnormal immune system response, rather than by infection. It is a chronic disease that requires long-term management. Symptoms include abdominal pain, diarrhea sometimes with blood, fatigue, weight loss, etc. These symptoms usually last for a long time and recur repeatedly. IBD may also affect other body parts, such as joints, skin, and eyes. Diagnostic methods mainly include endoscopic examination such as colonoscopy, imaging examination, and biomarkers such as fecal calprotectin. Treatment includes medications such as anti-inflammatory drugs, immunosuppressants, biologics, and in some cases surgery. Treatment is usually long-term and aims to control symptoms and reduce inflammation. Infectious enteritis is an acute intestinal inflammation caused by bacterial, viral, parasitic, and other infections. It is usually short-term and symptoms improve as the infection clears. Symptoms include sudden abdominal pain, diarrhea, vomiting, fever, etc. Symptoms appear quickly and usually last for a few days to a week. Fever is a common symptom, especially in bacterial infections. Diagnosis is mainly determined by specific pathogens through stool examination, blood examination, and other laboratory tests. In most cases, infectious enteritis will heal on its own. Treatment may include fluids and electrolytes to prevent dehydration, and antibiotics or antiviral drugs if necessary. | Although both IBD and infectious enteritis affect the intestine and cause similar symptoms, their causes and characteristics are different. IBD is a chronic inflammation caused by an abnormal immune system response, rather than by infection. This is a disease that requires long-term management, with symptoms including abdominal pain, diarrhea sometimes with blood, fatigue, and weight loss, which usually last for a long time and recur repeatedly. IBD can also affect other parts of the body, such as the joints, skin, and eyes. Diagnosis is mainly made through endoscopic examination such as colonoscopy, imaging examination, and biomarkers such as fecal calprotectin. Treatment modalities include medications such as anti-inflammatory drugs, immunosuppressants, biologics, and in some cases surgery, and often require long-term treatment to control symptoms and reduce inflammation. | Although IBD and infectious enteritis can both cause intestinal problems and lead to similar symptoms, their causes and characteristics are different. IBD is a chronic inflammation caused by an abnormal immune system response, rather than by infection. This is a long-term illness that requires ongoing management. Symptoms include abdominal pain, diarrhea sometimes with blood, fatigue, and weight loss, which often persist for a long time and recur repeatedly. IBD may also affect other body parts, such as the joints, skin, and eyes. Diagnostic methods mainly rely on endoscopic examination such as colonoscopy, imaging examination, and biomarkers such as fecal calprotectin. Treatment includes the use of medications such as anti-inflammatory drugs, immunosuppressants, biologics, and in some cases surgery, usually long-term, aimed at controlling symptoms and reducing inflammation. | There are significant differences between IBD and infectious enteritis in terms of etiology, symptoms, diagnosis, and treatment methods. Firstly, IBD is a chronic, nonspecific intestinal inflammatory disease of unknown cause, including Crohn's disease and ulcerative colitis. Infectious enteritis is usually caused by bacterial, viral, parasitic, or fungal infections, with the main route of transmission being fecal-oral transmission, often triggered by improper diet. Secondly, IBD patients often suffer from symptoms such as diarrhea, abdominal pain, and blood in the stool. In terms of diagnosis, IBD requires a comprehensive use of medical history, physical examination, laboratory tests, and imaging tests to make a judgment. Infectious enteritis is primarily diagnosed through clinical history and symptoms, and then confirmed through relevant tests. Finally, the treatment of IBD mainly relies on anti-inflammatory drugs, immunosuppressants, and hormones. Severe cases may require surgical intervention, while infectious enteritis is mainly treated with symptomatic treatment and lifestyle adjustments to alleviate symptoms, such as supplementing water and electrolytes and adjusting diet structure. | Firstly, from the perspective of etiology and pathogenesis, the causes of IBD are not fully understood, but it is known that abnormal responses of the intestinal mucosal immune system play a key role in its pathogenesis, involving the interaction of multiple factors such as environment, genetics, infection, and immunity. Infectious enteritis is mainly caused by bacteria, viruses and other microorganisms, and its pathogenesis is the inflammatory response caused by the invasion of pathogens into the intestinal mucosa. Secondly, in clinical manifestations, the symptoms of IBD are diverse, including diarrhea, abdominal pain, bloody stool, weight loss, fatigue, and so on. These symptoms may occur repeatedly. In contrast, the main symptoms of infectious enteritis are abdominal pain, diarrhea, nausea, vomiting, and in severe cases, complications such as fever and dehydration may occur. It is worth noting that ulcerative colitis in IBD is characterized by bloody diarrhea, while the diarrhea symptoms of Crohn's disease are relatively mild. In terms of treatment, the treatment of IBD involves various means such as general treatment, drug therapy, and surgical treatment. Drug therapy includes the use of anti-inflammatory drugs, immune regulatory drugs, and nutritional support drugs. The treatment of infectious enteritis mainly depends on antibiotics and other anti-infective drugs, as well as fluid replacement support measures. | The differences between IBD and infectious enteritis mainly lie in the causes, pathogenesis, clinical manifestations, and treatment methods. The etiology of IBD is not fully understood, and may be related to multiple factors such as genetics, immunity, environment, and infection. Infectious enteritis is mainly caused by microorganisms such as bacteria and viruses, and is a clear intestinal infectious disease caused by pathogens. IBD mainly manifests as diarrhea, abdominal pain, bloody stool, weight loss, fatigue, and other symptoms. The severity of the condition varies and it is prone to recurrent attacks. The main symptoms of infectious enteritis are abdominal pain, diarrhea, nausea, vomiting, etc., which may be accompanied by fever. There are various treatments for IBD, including anti-inflammatory drugs, immune-regulating drugs, probiotic therapy, and so on the main treatment for infectious enteritis is antibiotic anti-infection, fluid infusion support, and treatment of the pathogen. |
| 3. Why do I need to undergo so many tests to confirm the diagnosis of IBD? | Before confirming IBD, many tests are needed to ensure the accuracy of the diagnosis, rule out other possible diseases, and fully understand the severity of the condition. Many other diseases can cause symptoms similar to IBD, such as abdominal pain, diarrhea, and weight loss. Common conditions include infectious enteritis, irritable bowel syndrome, celiac disease, and early colon cancer. Secondly, IBD includes Crohn's disease and ulcerative colitis. Although the symptoms of both are similar, they affect the gut in different ways and are treated differently. Several tests can help determine which type is present. Third, understanding the severity of the condition helps to develop appropriate treatment plans. Different examinations can provide information about the degree of inflammation, the location of the lesion, and the presence of complications. | Before confirming IBD, many tests are needed to ensure the accuracy of the diagnosis, rule out other possible diseases, and fully understand the severity of the condition. Many other diseases can cause symptoms similar to IBD, such as abdominal pain, diarrhea, and weight loss. Common conditions include infectious enteritis, irritable bowel syndrome, celiac disease, and early colon cancer. Secondly, IBD includes Crohn's disease and ulcerative colitis. Although the symptoms of both are similar, they affect the gut in different ways and are treated differently. Various tests can help determine which type it is. Third, understanding the severity of the condition helps to develop appropriate treatment plans. Different tests can provide information about the degree of inflammation, the location of the lesion, and whether there are complications. | Before confirming IBD, many tests are needed to ensure the accuracy of the diagnosis, rule out other possible diseases, and fully understand the severity of the condition. Many other diseases can cause symptoms similar to IBD, such as abdominal pain, diarrhea, and weight loss. Common conditions include infectious enteritis, irritable bowel syndrome, celiac disease, and early colon cancer. In addition, IBD includes Crohn's disease and ulcerative colitis. Although the symptoms of these two diseases are similar, they affect the gut in different ways and have different treatment methods. Various tests can help determine which type it is. Finally, understanding the severity of the condition helps to develop appropriate treatment plans. Different tests can provide important information about the degree of inflammation, the location of the lesion, and the presence of complications. | IBD is a disease that affects the entire gastrointestinal tract, including the esophagus, stomach, duodenum, small intestine, and large intestine, and its main manifestation is gastrointestinal ulceration. However, this type of ulceration is often non-specific and therefore lacks clear diagnostic criteria. It requires the exclusion of other possibilities and multiple diagnostic methods to make a definitive diagnosis. Therefore, it is necessary to combine other evidence for diagnosis, especially for diseases such as Crohn's disease and intestinal tuberculosis, which are easily confused and need to be gradually corrected during the treatment process. In addition, the symptoms of IBD are similar to those of other diseases, such as infectious enteritis or drug-induced colitis, which can lead to symptoms such as pain, cramps, and diarrhea. Therefore, colonoscopy is difficult to diagnose accurately, and pathological results are often atypical, mainly used to rule out other diseases. | IBD patients need to undergo many tests because the symptoms of IBD are not typical and are similar to those of chronic gastroenteritis, intestinal tuberculosis, biliary tract disease, chronic bacterial dysentery, and pancreatitis. There is a lack of gold standard for diagnosis, and in order to make a definitive diagnosis, it is necessary to rule out other diseases one by one and use many diagnostic methods and tools. Moreover, some IBD patients have lesions that involve multiple sites, and sometimes the required tests for different sites of lesions are different, which can also lead to an increase in the number of tests required for patients to diagnose IBD. Finally, a comprehensive examination can fully understand the severity of IBD and help patients develop appropriate treatment plans. | The clinical manifestations of IBD are diverse, such as diarrhea, abdominal pain, bloody stool, etc., and the severity of the disease varies, with frequent relapses. It is necessary to differentiate it from other similar diseases through various means such as physical examination, endoscopy, laboratory examination, and imaging examination. Through physical examination, doctors can preliminarily determine whether the patient has abdominal pain, tenderness, and the location of the pain, which helps to judge the severity of the condition; Endoscopic examination can visually observe changes in the intestinal wall, such as continuous or discontinuous changes, as well as the specific conditions of the lesion site; Laboratory tests such as routine stool examination can provide information about the characteristics and composition of stool, which can help assist in diagnosis; Imaging tests such as CT and magnetic resonance imaging can reflect the condition of the intestinal wall, which is helpful in determining the location of the lesion and whether there is stenosis in the intestine. |
| 4. What is the difference between ulcerative colitis and Crohn's disease? | Although ulcerative colitis and Crohn's disease are both IBD, they have significant differences in symptoms, endoscopic findings, complications, and pathology. In terms of symptoms, ulcerative colitis is mainly characterized by diarrhea with blood and mucus, abdominal pain, rectal bleeding, urgent need to defecate, and frequent bowel movements. The lesion site is often limited to the colon and rectum, and inflammation usually begins in the rectum and spreads upward in a continuous distribution. The main symptoms of Crohn's disease are abdominal pain, usually in the lower right abdomen, diarrhea without blood, weight loss, fever, and fatigue. The location of the lesion can affect the entire digestive tract, from the mouth to the anus, but it is most commonly found in the terminal ileum and colon. Inflammation is segmentally distributed, with alternating areas of disease and normal tissue. From the endoscopic findings, ulcerative colitis is a continuous inflammation that extends upward from the rectum, with lesions characterized by redness, bleeding, erosion, and ulceration of the mucosa. In severe cases, pseudopolyp and pus can be seen. Crohn's disease often presents as jumping lesions, longitudinal ulcers, fissured ulcers, intestinal wall thickening, and stenosis under endoscopy. The appearance of "pebble-like" can be seen. In terms of complications, ulcerative colitis can be associated with severe bleeding, toxic megacolon, perforation, and an increased risk of colon cancer. Crohn's disease Crohn's disease intestinal complications can be accompanied by fistula, abscess, intestinal obstruction, malnutrition, and increased risk of small intestinal cancer. From the perspective of intestinal biopsy pathology, ulcerative colitis mainly affects the mucosal and submucosal layers, with pathological characteristics of mucosal inflammation, crypt abscesses, flattened mucosa, and pseudopolyps. Crohn's disease is a full-thickness inflammation, affecting all layers from the mucosa to the serosa. The pathological features include granulomas, fissured ulcers, lymphatic aggregation, fibrosis, and intestinal wall thickening. | Although both ulcerative colitis and Crohn's disease are forms of IBD, they exhibit notable differences in symptoms, endoscopic presentations, complications, and pathology. The main symptoms of ulcerative colitis include diarrhea often accompanied by blood and mucus, abdominal pain, rectal bleeding, urgency and frequency of bowel movements. The lesion is usually confined to the colon and rectum, with inflammation starting in the rectum and spreading upwards in a continuous distribution. The main symptoms of Crohn's disease are abdominal pain, usually in the lower right abdomen, diarrhea that may not be bloody, weight loss, fever, and fatigue. The disease can affect the entire digestive tract, from the mouth to the anus, but is most common in the terminal ileum and colon. Inflammation is segmentally distributed, with alternating areas of disease and normal tissue. Under endoscopy, ulcerative colitis is characterized by continuous inflammation that begins in the rectum and spreads upward, with lesions showing redness, bleeding, erosion, and ulceration of the mucosa. In severe cases, pseudopolyp and pus can be seen. The endoscopic manifestations of Crohn's disease include jumping lesions, longitudinal ulcers, fissured ulcers, intestinal wall thickening, and stenosis, with a "pebble-like" appearance. In terms of complications, ulcerative colitis may lead to severe bleeding, toxic megacolon, perforation, and an increased risk of colon cancer. Complications of Crohn's disease include fistulas, abscesses, intestinal obstruction, malnutrition, and an increased risk of small intestinal cancer. Pathologically, ulcerative colitis mainly affects the mucosal and submucosal layers, characterized by inflammation of the mucosal layer, crypt abscesses, flattening of the mucosa, and pseudopolyp formation. Crohn's disease is a full-thickness inflammation that affects all layers from the mucosa to the serosa, with pathological features including granulomas, fissured ulcers, lymphoid aggregates, fibrosis, and intestinal wall thickening. | Although ulcerative colitis and Crohn's disease belong to IBD, they have significant differences in symptoms, endoscopic findings, complications, and pathology. Symptoms of ulcerative colitis include diarrhea often accompanied by blood and mucus, abdominal pain, rectal bleeding, urgency, and frequency of bowel movements. The lesions are usually limited to the colon and rectum, with inflammation starting in the rectum and spreading upward in a continuous distribution. Symptoms of Crohn's disease include abdominal pain, usually in the lower right abdomen, diarrhea that may not be bloody, weight loss, fever, and fatigue. The lesions can involve the entire digestive tract, from the mouth to the anus, but are most commonly found in the terminal ileum and colon, with inflammation distributed in segments, alternating between diseased and normal areas. In endoscopic examination, ulcerative colitis is manifested as continuous inflammation that extends upward from the rectum, with lesions showing redness, bleeding, erosion, and ulceration of the mucosa. In severe cases, pseudopolyp and pus can be seen. The endoscopic manifestations of Crohn's disease include jumping lesions, longitudinal ulcers, fissured ulcers, thickening and narrowing of the intestinal wall, and a "pebble-like" appearance. Complications of ulcerative colitis may include severe bleeding, toxic megacolon, perforation, and an increased risk of colon cancer. Complications of Crohn's disease include fistulas, abscesses, intestinal obstruction, malnutrition, and an increased risk of small intestinal cancer. Pathologically, ulcerative colitis mainly affects the mucosal and submucosal layers, characterized by inflammation of the mucosal layer, crypt abscesses, flattening of the mucosa, and pseudopolyps. Crohn's disease affects the entire intestinal tract, with inflammation from the mucosa to the serosa. Pathological features include granulomas, fissured ulcers, lymph node accumulation, fibrosis, and thickening of the intestinal wall. | Although Crohn's disease and ulcerative colitis share some similar symptoms, there are still some significant differences between the two. For example, the inflammation of Crohn's disease can accumulate in all digestive organs from the mouth to the anus, including the esophagus, stomach, small intestine, and large intestine, while ulcerative colitis is usually limited to the large intestine, namely the colon and rectum; For another example, the inflammation of Crohn's disease is "segmental", which means that it only affects a part of the intestine, with normal tissue between the affected areas. In contrast, the inflammation of ulcerative colitis begins in the rectum and gradually spreads upwards in a continuous manner. | There are significant differences in the location, clinical manifestations, and degree of harm between ulcerative colitis and Crohn's disease. Firstly, ulcerative colitis mainly affects the mucosal and submucosal layers of the colon, starting from the anal end of the rectum and affecting the sigmoid colon and descending colon, without involving the small intestine. Crohn's disease can occur anywhere along the entire digestive tract, from the mouth to the rectum, especially in the terminal ileum and right colon. Secondly, the typical symptoms of ulcerative colitis include abdominal pain, diarrhea, mucus-rich bloody stool, and a sense of urgency and tenesmus. Although Crohn's disease also involves diarrhea, it does not present with bloody stool or a sense of urgency and tenesmus. | The difference between ulcerative colitis and Crohn's disease generally lies in the location of the disease, the extent of the lesion, the depth of the lesion, the clinical manifestations, and the treatment methods. 1. Location of onset: Ulcerative colitis generally occurs in the sigmoid colon and rectum, while Crohn's disease usually occurs in any part of the gastrointestinal tract. 2. Extent of the lesion: Ulcerative colitis typically affects only the colon, without affecting the small intestine or upper digestive tract. Crohn's disease may affect the entire digestive tract. 3. Clinical manifestation: Ulcerative colitis may generally lead to diarrhea, abdominal pain, etc., while Crohn's disease may lead to abdominal pain, diarrhea, weight loss, and other symptoms. 4. Characteristics of biopsy: Diffuse inflammation in the full thickness of the lamina propria of ulcerative colitis, crypt abscesses, obvious abnormalities in crypt structure, decreased goblet cells, and Crohn's disease with fissured ulcers, non-caseating granulomas, and submucosal lymphocyte aggregation. 6 Endoscopic findings: Ulcerative colitis with shallow ulcers, diffuse congestion and edema of the mucosa, granular appearance, increased fragility, and Crohn's disease with longitudinal ulcers and pebble-like appearance. The appearance of the mucosa between lesions is normal (not diffuse) |
| 5. What are the clinical symptoms of IBD? | The symptoms of IBD vary from person to person, and may range from mild to severe. Common symptoms of ulcerative colitis include diarrhea often with blood and mucus, abdominal pain often located in the lower left abdomen, often accompanied by a sense of urgency and discomfort before defecation, weight loss and fatigue, anemia, and so on; Common symptoms of Crohn's disease include abdominal pain, usually in the lower right abdomen, sometimes extending to the entire abdomen, diarrhea, weight loss, fatigue, fever, and perianal lesions such as anal fissures, anal fistulas, and abscesses. At the same time, both of them will also have symptoms such as loss of appetite, joint pain and arthritis, skin erythematous nodules, iritis and scleritis, and primary sclerosing cholangitis. | The symptoms of IBD vary from person to person, and may present in varying degrees of severity. Common symptoms of ulcerative colitis include diarrhea often with blood and mucus, abdominal pain often located in the lower left abdomen, often accompanied by a sense of urgency and discomfort before defecation, weight loss, fatigue, and anemia. Common symptoms of Crohn's disease include abdominal pain, usually in the lower right abdomen, sometimes extending to the entire abdomen, diarrhea, weight loss, fatigue, fever, and perianal lesions such as anal fissures, anal fistulas, and abscesses. In addition, both may present with loss of appetite, joint pain and arthritis, skin red nodules, iritis and scleritis, and primary sclerosing cholangitis. | The symptoms of IBD can vary from person to person, and may vary in severity. Common symptoms of ulcerative colitis include diarrhea often with blood and mucus, abdominal pain in the lower left abdomen often accompanied by urgency and discomfort before defecation, weight loss, fatigue, and anemia. Common symptoms of Crohn's disease include abdominal pain in the lower right abdomen, which may sometimes extend to the entire abdomen, diarrhea, weight loss, fatigue, fever, and perianal lesions such as anal fissures, anal fistulas, and abscesses. In addition, both diseases may lead to symptoms such as loss of appetite, joint pain and arthritis, skin red nodules, iritis and scleritis, and primary sclerosing cholangitis. | IBD can produce a variety of clinical manifestations, including but not limited to the following: digestive symptoms such as diarrhea, abdominal pain, hematochezia, perianal abscess, etc; Systemic symptoms such as weight loss, fever, fatigue, anemia, etc; Parenteral manifestations such as joint, skin, mucosa, eye lesions, etc; In severe cases, intestinal perforation, intestinal obstruction, massive bleeding, and even canceration may occur. In addition to affecting the intestine, IBD may also cause a variety of extraintestinal manifestations, including skin symptoms such as rashes and ulcerative stomatitis, arthritis, eye problems such as conjunctivitis and scleritis, liver problems, and so on. | IBD, including ulcerative colitis and Crohn's disease, exhibit different symptoms. The intestinal symptoms of ulcerative colitis include diarrhea, bloody and purulent stool, abdominal pain, nausea, vomiting, and tenesmus. At the same time, patients may have systemic symptoms such as fever, fatigue, anemia, and malnutrition, as well as extraintestinal symptoms such as oral ulcers, eye disease, arthritis, and liver disease. Crohn's disease may manifest as sudden diarrhea, abdominal pain, especially in the lower right abdomen, accompanied by bloody stools. In addition, patients may experience systemic symptoms such as fatigue, weight loss, fever, anemia, malnutrition, as well as oral ulcers, eye disease, arthritis, skin redness and pain, usually in the legs, and liver disease. In addition, there may be extraintestinal symptoms such as arthritis and conjunctivitis. | Ulcerative colitis most commonly occurs in young adults, with clinical manifestations of persistent or recurrent diarrhea, mucous purulent bloody stool with abdominal pain, tenesmus, and varying degrees of systemic symptoms. The course of disease is usually more than 4-6 weeks, and it can also be accompanied by extraintestinal symptoms such as skin, mucous membrane, joints, eyes, liver and gallbladder manifestations. Crohn's disease is more common in people aged 18-35, with slightly more males than females. The symptoms are diverse, including diarrhea, abdominal pain, weight loss, fever, anemia, etc. Adolescents may have growth retardation, and extraintestinal manifestations are similar to ulcerative colitis. Complications are common, including fistula, abdominal abscess, intestinal stenosis and obstruction, perianal lesions, etc. Less common complications include massive gastrointestinal bleeding, intestinal perforation, and canceration in long-term patients. Note that young patients with diarrhea, abdominal pain, weight loss, especially with extraintestinal manifestations or perianal lesions, should be highly suspected of having this disease. |
| 6. Why do I often feel tired? | Feeling tired is one of the common problems for IBD patients, but there are several possible causes. First, the disease itself may cause the body to be in a persistent inflammatory state, which may drain your energy. Secondly, if your intestines are in a state of inflammation or damage, the body may not be able to absorb enough nutrients, which can also lead to fatigue. In addition, some medications used in drug therapy may also cause fatigue, as they may affect sleep quality or have other effects on the body. Finally, long-term emotional stress and anxiety in dealing with illness may also exacerbate feelings of fatigue. | Feeling tired is one of the common problems for IBD patients, and there are several reasons for it. Firstly, the disease itself may lead to a persistent inflammatory state in the body, consuming a lot of energy. Secondly, inflammation or damage to the intestine may affect nutrient absorption, leading to malnutrition and fatigue. In addition, certain medications may also cause fatigue because they may affect sleep quality or produce other side effects. Finally, long-term emotional stress and anxiety in dealing with illness can also exacerbate feelings of fatigue. | IBD patients often feel tired, which may have multiple causes. Firstly, the disease itself can put the body in a state of persistent inflammation, consuming a lot of energy. Secondly, inflammation or damage to the intestine may lead to poor absorption of nutrients, which can cause fatigue. In addition, some therapeutic drugs may cause fatigue because they may affect sleep or produce other side effects. Finally, long-term coping with emotional stress and anxiety caused by illness can also exacerbate fatigue. | The reasons why IBD patients often feel tired are multifaceted, including long-term diarrhea, malnutrition, anemia, fever, and inflammation. The reasons why IBD patients often feel tired can be summarized as follows: 1. Long-term diarrhea leads to a disturbance in the balance of water and electrolytes in the patient's body, which consumes a lot of energy and leads to physical fatigue. Malnutrition: IBD may lead to intestinal absorption disorders and excessive consumption, making it difficult for patients to absorb enough nutrients from food. This malnutrition state can lead to weight loss, anemia, hypoproteinemia, and further exacerbate the body's fatigue. 3. Anemia: Patients with IBD often have mild anemia, but during acute attacks, due to massive bleeding, it may lead to severe anemia, causing patients to feel fatigue and weakness. Fever and inflammation: Patients with IBD may experience fever and systemic inflammatory reactions, which can consume the patient's energy and make them feel tired. | Firstly, IBD patients often suffer from diarrhea, which can lead to water and electrolyte imbalance, especially in patients with ulcerative colitis, where the number of daily diarrhea episodes can be as high as 10 to 30. This persistent diarrhea can lead to a disturbance in the balance of water and electrolytes in the patient's body, which can subsequently cause fatigue. Secondly, due to intestinal inflammation affecting the absorption of nutrients, patients often experience symptoms such as malnutrition and anemia. Malnutrition can lead to insufficient energy supply to the body, while anemia can affect the oxygen-carrying capacity of the blood, both of which work together to make patients feel tired. Thirdly, IBD may lead to systemic symptoms such as fever and systemic inflammatory response, which not only consume the patient's energy but also may affect sleep quality, further exacerbating fatigue. Fourth, patients who have been plagued by diseases for a long time may face greater psychological and mental pressure, which can also make them feel exhausted. | The fatigue experienced by IBD patients is a result of a combination of factors, including the impact of the disease itself, inflammatory responses, psychosocial factors, side effects of medications, and other factors. IBD can lead to impaired intestinal function and affect the absorption of nutrients, which can lead to malnutrition, anemia, and other symptoms over time, causing fatigue. Symptoms such as diarrhea and abdominal pain can cause patients to consume more energy, leading to a decrease in physical strength and a sense of fatigue. IBD often has recurrent attacks and unstable conditions, which can cause significant psychological stress for patients, such as anxiety and depression. These mental and psychological factors can also exacerbate the patient's sense of fatigue. Drugs for treating IBD, such as immunosuppressants and antibiotics, may have some side effects, such as fatigue and tiredness. |
| 7. What are the extra-intestinal manifestations of IBD? | IBD patients may have a variety of extraintestinal manifestations that affect joints, skin, eyes, liver, kidneys, and other systems, including joint lesions such as peripheral arthritis and ankylosing spondylitis; Skin lesions, such as erythematous nodules and gangrenous pyoderma; Eye disorders such as iritis, scleritis, and conjunctivitis; Liver and biliary tract diseases, such as primary sclerosing cholangitis, fatty liver, etc; oral lesions, such as oral ulcers; Abnormal cardiovascular system, such as deep vein thrombosis. | IBD not only affects the intestine, but also causes a variety of extraintestinal manifestations, which may include joint pain and arthritis, skin problems such as erythematous nodules and gangrenous pyoderma, eye diseases such as iritis and scleritis, and liver diseases such as primary sclerosing cholangitis. In addition, patients may also experience problems such as oral ulcers, osteoporosis, and kidney stones. The appearance of extraintestinal manifestations is often related to the activity level of intestinal diseases, but it may also exist independently. | The extraintestinal manifestations of IBD include joint pain and arthritis, skin lesions such as erythematous nodules and gangrenous pyoderma, eye diseases such as iritis and scleritis, and liver problems such as primary sclerosing cholangitis. Patients may also experience oral ulcers, osteoporosis, and kidney stones, which are closely related to the activity level of intestinal diseases and sometimes exist independently. | The extraintestinal manifestations of IBD mainly involve multiple systems such as the skin, oral cavity, skeletal muscle system, eyes, liver and gallbladder, and blood. Joint symptoms are common extraintestinal manifestations of IBD, mainly including peripheral arthritis, central arthritis, isolated arthritis, osteoporosis, and osteomalacia. Some IBD patients have oral mucosal lesions as the initial symptom, including aphthous ulcer, proliferative suppurative stomatitis, gingivitis, and cheilitis. The most common cutaneous manifestations are erythema nodosum and pyoderma gangrenosum, which may also be associated with psoriasis and Sweet's syndrome. The incidence of ocular manifestations in IBD patients is relatively low, including superficial scleritis, anterior uveitis, conjunctivitis, iritis, etc. | The extraintestinal manifestations of IBD mainly include erythema nodosum, pyoderma gangrenosum, oral lesions, bone and joint lesions, liver and gallbladder lesions, eye diseases, and cardiovascular system diseases. The two most common types of lesions are joint disease and eye disease. Joint disease is one of the most common off-site manifestations of IBD, and some IBD patients may experience symptoms of sacroiliitis. | The extraintestinal manifestations of IBD include joint damage (such as peripheral arthritis, spondyloarthritis, etc.), skin and mucous membrane manifestations (such as oral ulcers, erythema nodosum, and gangrenous pyoderma), eye lesions (such as iritis and scleritis), liver and gallbladder diseases (such as fatty liver, primary sclerosing cholangitis, and cholelithiasis), thromboembolic diseases, etc. |
| 8.Will I turn into colorectal cancer? | IBD patients, especially those with ulcerative colitis and Crohn's disease affecting the colon, do have a higher risk of colon cancer. However, the specific development of colon cancer risk varies from individual to individual. The longer you have IBD, the higher your risk of colon cancer. Typically, after 8-10 years of illness, the risk significantly increases. The wider the inflammation of ulcerative colitis, the higher the risk. Patients with generalized colitis involving the entire colon are at a higher risk than those with colitis limited to the rectum or left colon. Patients with Crohn's disease affecting the colon are also at higher risk, especially those with extensive involvement of the colon. Long-term severe intestinal inflammation increases the risk of colon cancer. Chronic active inflammation and persistent lesions are more likely to undergo cancerization. If someone in the family has colon cancer, the risk of cancer in the individual will also increase. | IBD, which includes ulcerative colitis and Crohn's disease, does increase the risk of colorectal cancer, which is mainly related to long-term chronic intestinal inflammation. In ulcerative colitis, inflammation is usually confined to the colon and rectum, and as the duration of the disease increases, especially after 8-10 years, the risk of cancer significantly increases. If the inflammation is widespread, especially in patients with involvement of the entire colon, the risk of cancer is higher. In addition, the severity and frequency of inflammation are also decisive factors, and patients with frequent acute attacks and high activity of inflammation are more likely to develop cancer. Patients with Crohn's disease who only have small intestinal involvement have a relatively low risk of colorectal cancer, but when Crohn's disease affects the colon, the risk increases significantly. Similar to ulcerative colitis, long-term chronic inflammation and extensive intestinal involvement are major risk factors. Chronic inflammation leads to repeated injury and repair of intestinal mucosal cells. This ongoing process of inflammation and cell regeneration may cause DNA damage and mutations, thereby increasing the likelihood of cancer. | IBD, including ulcerative colitis and Crohn's disease, does increase the risk of developing colorectal cancer. This risk is mainly related to chronic, long-term intestinal inflammation. For ulcerative colitis, inflammation is usually limited to the colon and rectum, while Crohn's disease may affect the entire digestive tract, but when Crohn's disease involves the colon, it also increases the risk of colorectal cancer. Chronic inflammation can lead to repeated injury and repair processes of intestinal mucosal cells. This sustained inflammation and cell regeneration process may cause DNA damage and mutations in cells, thereby increasing the likelihood of cancerization. The risk of cancer in patients with ulcerative colitis increases with the duration of the disease, usually after 8-10 years of illness, with a significant increase in risk. If the lesion is extensive, especially involving the entire colon, the risk is higher. In addition, the activity and severity of the disease are also one of the determining factors. Patients with frequent acute attacks and high activity of inflammation have a greater risk of canceration. For patients with Crohn's disease, if the inflammation is limited to the small intestine, the risk of colorectal cancer is relatively low. However, when Crohn's disease affects the colon, the risk of cancer significantly increases. Similar to ulcerative colitis, long-term chronic inflammation and extensive intestinal involvement are major risk factors. | Patients with a longer duration of IBD do have a higher risk of developing colorectal cancer than the general population. Some studies have pointed out that the incidence of colorectal cancer begins to increase about 10 years after the diagnosis of IBD, and the longer the duration of the disease, the higher the probability of developing cancer. Crohn's disease is more likely to occur in the small intestine, and patients with Crohn's disease have a higher risk of developing small intestinal tumors than patients with ulcerative colitis; In addition, long-term anal and perianal lesions are high-risk factors for anal cancer in patients with Crohn's disease, so adequate attention should be paid to chronic perianal lesions. The younger age of initial onset and extensive colonic lesions in IBD patients are risk factors for cancerization; In the case of primary sclerosing cholangitis (PSC), the risk of canceration is significantly increased, and the risk of developing bile duct cancer in such patients is also increased; IBD patients with a family history of colorectal cancer are twice as likely to develop cancer as those without a family history. | Ulcerative colitis may indeed become cancerous. Ulcerative colitis is a chronic nonspecific inflammatory disease of the intestine, and its risk of canceration varies from individual to individual, and is generally closely related to the duration of the disease. Research shows that the longer the disease course, the greater the risk of canceration. Patients with a disease duration of more than 10 years have a significantly increased risk of canceration. If there is a family history of colorectal cancer, an individual's risk of developing cancer also increases. Cancerization mainly occurs in the colon, and areas with chronic active inflammation and persistent lesions are more likely to undergo cancerization. Patients with extensive colon type and left colon type have a higher risk of canceration, while patients with rectal type have a relatively lower risk of canceration. | Patients with ulcerative colitis have a certain risk of canceration, but not all patients will develop cancer. The risk of canceration is related to many factors, including genetics, environment, immunity and other factors. Especially when the patient has a long history of disease, such as 8 years or more, special vigilance is needed to prevent canceration. The canceration rate of ulcerative colitis varies depending on the severity of the disease and treatment conditions. According to different research reports, the canceration rate is roughly between 0.7% and 8%, and in severe cases, it may even reach about 13%. The intestinal mucosa of patients with long-term chronic ulcerative colitis may undergo a process of continuous proliferation, repair, and damage of some mucosa under the stimulation of chronic inflammation. During this process, some intestinal mucosa may undergo mutations, thereby increasing the risk of cancerization. |
| 9. Will IBD have an impact on my life expectancy? | Current research suggests that IBD itself usually does not directly lead to a shorter life span. However, IBD may increase some health-related risk factors, which can affect life expectancy. First, IBD may increase the risk of other health problems. For example, people with IBD may be more likely to develop other chronic diseases such as cardiovascular disease, osteoporosis, and cancer, which can affect longevity. Secondly, IBD patients may need long-term medication, such as immunosuppressants and biologics, to control inflammation and maintain remission. These medications may increase certain health risks, such as the risk of infections and cancer, especially in the case of long-term use. Additionally, IBD may negatively affect quality of life, including emotional and mental health aspects. Long-term emotional stress and anxiety in dealing with illness may affect physical health and thus affect life expectancy. | Having IBD does not necessarily significantly shorten life expectancy, but it does have an impact on quality of life and carries a certain risk of complications. Both ulcerative colitis and Crohn's disease are chronic conditions that require long-term management and treatment. With the advancement of medical technology and drugs, many patients can effectively control their symptoms and maintain a normal life. However, IBD patients may face some serious complications, such as intestinal obstruction, perforation, toxic megacolon, severe bleeding, and increased risk of bowel cancer, which may affect the prognosis. In addition, repeated attacks of the disease and long-term drug use may also lead to other health problems, such as increased risk of infection, osteoporosis, liver disease, and so on. Overall, although IBD may have an impact on life expectancy, many patients can lead a relatively normal and healthy life through active disease management, regular monitoring, and appropriate treatment. | After developing IBD, the life expectancy of patients usually does not significantly decrease, especially if they receive appropriate treatment and good management. However, IBD does affect quality of life and may increase the risk of some complications, such as intestinal obstruction, perforation, toxic megacolon, and colorectal cancer, which can affect prognosis in some cases. In addition, the chronic nature and repeated attacks of the disease can cause distress to the patient's daily life, and long-term use of certain drugs may also bring side effects, such as increased risk of infection and osteoporosis. Nonetheless, with the advancement of medical technology and treatment methods, many patients can effectively control their symptoms and lead relatively normal lives. | Most statistics show that IBD does not have a particular impact on the life span of patients. A previous study based on 20 years of follow-up in Europe after the diagnosis of Crohn's disease also showed that the disease has no significant difference in life expectancy and the probability of death due to cancer and cardiovascular disease compared to the general population. In addition, many cases also show that as long as the condition of IBD patients is well controlled, many patients can have a fulfilling and meaningful life. | Statistics show that IBD has no special impact on the life expectancy of patients. A study based on 20 years of follow-up in Europe after the diagnosis of Crohn's disease also showed that the disease has no significant difference in life expectancy and the probability of death due to cancer and cardiovascular disease compared to the general population. In addition, many cases also show that as long as the disease is well controlled, many patients can have a fulfilling and meaningful life. However, if the treatment is not appropriate or no treatment is given, some complications such as bleeding, perforation, and intestinal fistula may occur, which can be life-threatening. | The life span of IBD patients is affected by many factors, and there is no clear clinical data to support accurate prediction of their life span. Generally, IBD does not directly affect the life span of patients, but it has a potential impact on life span through its impact on quality of life, complications, and treatment and nutrition, including disease activity, complications, treatment, nutrition, and mental health. The key is to maximize life expectancy and quality of life through effective medical management, following doctors' advice, maintaining a healthy lifestyle, and regular follow-ups. |
| 10. Will IBD be passed on to my children? | IBD may be related to genetic factors, but genetic factors are not the only influencing factor. The incidence rate of IBD in the general population is approximately 0.1-0.2%. If one parent has IBD, the risk of the child developing the disease increases to approximately 2-9%. If both parents have IBD, the risk of the child developing the disease is higher, possibly reaching 30-50%. Although IBD may occur in some families, it is not entirely determined by genes. Genetic factors may increase the risk of developing IBD, but environmental factors may also play an important role in its onset. Studies have shown that certain genetic variations are associated with the onset of IBD, but these genetic variations are not sufficient to cause IBD by themselves, but rather are related to the interaction with environmental factors. For example, environmental factors such as diet, smoking, infections and gut microbes may play a major role in people with IBD. | IBD has a certain genetic predisposition, and the risk of children with IBD is relatively high for parents who have IBD. Although the specific genetic mechanism has not been fully clarified, studies have shown that genes play an important role in the pathogenesis of IBD. If you have IBD, your child is at a higher risk of developing the disease than the general population. However, genetic factors are only one part of the causes of the disease, and environmental factors, immune system response, and other unknown factors also play a key role in the pathogenesis. So, despite the genetic risk, not all children of parents with IBD will get the disease. | IBD has a genetic predisposition. If you have IBD, your child's risk of developing the disease is higher than that of the general population. Research has shown that genes play an important role in the pathogenesis of IBD, and scientists have discovered some gene variations associated with IBD. These genetic variations can affect the function of the immune system, causing the gut to overreact to normal bacteria or other environmental factors, leading to inflammation. However, genetic factors are only one aspect of IBD, and environmental factors also play an important role. Diet, infections, stress, and other lifestyle factors can all affect the onset of IBD. So while you have IBD, your child does not necessarily have to have this disease, but they do have a higher risk. Specifically, if one parent has IBD, the risk of the child developing the disease is about 2-13%, while if both parents have IBD, this risk increases significantly. Even so, not all children with genetic risk will develop the disease, as the occurrence of the disease usually requires the combined effects of multiple factors, including the interaction between genetic and environmental factors. | According to foreign data, the risk of IBD in offspring of parents with IBD is 2-13 times higher than that in the general population. If both parents have IBD, the risk of IBD in offspring is greater than 30%. Although IBD has a genetic predisposition, the overall incidence rate remains low. In addition, the risk of IBD is affected by many factors, including genetics, environment, lifestyle, immune response, and intestinal flora. In addition, IBD is a typical polygenic disease, and its pathogenesis involves the interaction of multiple genes and environmental factors. Currently, there is no method that can completely prevent or avoid the development of IBD. Through standardized treatment and disease control, IBD patients can still have healthy babies. | IBD can be passed on to children. IBD involves complex interactions between genetics, environment, immunity, and microbiota. Genetics does play a role in the onset of IBD. Clinical data show that the lifetime risk of first-degree relatives of patients with Crohn's disease is 5.2%, and the lifetime risk of first-degree relatives of patients with ulcerative colitis is 1.6%, which is much higher than that of the general population. Research has found that some genes increase the chance of developing IBD. More than 200 genes have been identified as being associated with the onset of IBD. From a genetic perspective, even if both parents do not have IBD, the patient may inherit both parents' susceptible genes at the same time, and there may be new gene mutations during the genetic process. In short, genetic factors can only increase the probability of developing IBD. Parents with IBD may not necessarily have children with IBD, and children of parents without IBD may also develop IBD. | IBD has genetic susceptibility, and generally speaking, there is a genetic tendency for IBD to occur clinically. The incidence rate of first-degree relatives of IBD patients is significantly higher than that of the general population, while the incidence rate of spouses of patients does not increase. IBD has a genetic predisposition. That is to say, the defects in genetic structure make some people more susceptible to IBD, which is manifested in racial differences, family clustering, and of course, the consistency of the co-occurrence of twins is significantly increased. If someone in the family has IBD, the probability of having IBD is higher than that of people without a family history. |
| **Treatment** | | | | | | |
| 1. Can hormones be used for a long time? | Long-term use of hormones may have many potential side effects and risks, including osteoporosis, diabetes, hypertension, increased risk of infection, and gastric mucosal injury. Long-term use of hormones can inhibit the function of the adrenal cortex, preventing the body from producing sufficient cortisol under stressful conditions. Therefore, it is usually not recommended as the main treatment for IBD in the long term. The use of hormones usually adopts the principle of short-term use and gradual reduction. In addition, by using immune modulators, biological agents, and other alternative treatments, combined with a healthy lifestyle and regular monitoring, IBD can be effectively managed and hormone dependence can be reduced. | Long-term use of corticosteroids such as prednisone or hydrocortisone is generally not recommended for IBD patients, although these drugs are very effective in controlling acute attacks. Long-term use of hormones can have many serious side effects, including osteoporosis, diabetes, hypertension, increased risk of infection, and bone necrosis. The side effects of hormones not only affect physical health, but also may have a negative impact on quality of life. Therefore, hormonal medications are usually used for short-term treatment to quickly control inflammation and symptoms. After that, doctors usually gradually reduce the dose of hormones and introduce other long-term maintenance treatment options, such as immunosuppressants or biologics, to maintain the remission state of the disease and reduce the risk of recurrence. This treatment strategy aims to balance disease control with drug side effects, ensuring patients can achieve optimal long-term health management outcomes. | Long-term use of steroids is generally not recommended for IBD patients, as although these drugs are very effective in controlling acute attacks, long-term use can cause many serious side effects, including osteoporosis, diabetes, hypertension, increased risk of infection, and osteonecrosis. Hormonal medications are typically prescribed for short-term treatment to swiftly manage inflammation and symptoms. After that, doctors usually gradually reduce the dose of hormones and introduce other long-term maintenance treatment options, such as immunosuppressants or biologics, to maintain the remission state of the disease and reduce the risk of recurrence. This treatment strategy aims to balance disease control with drug side effects, ensuring patients can achieve optimal long-term health management outcomes. | The use of IBD hormones requires caution, and long-term use is generally not recommended. Glucocorticoids can suppress the immune system to weaken the body's ability to produce and maintain inflammatory responses. Prednisone and prednisolone are used for moderate to severe diseases and ulcerative colitis. Budesonide may be used for mild to moderate Crohn's disease involving the ileum and right-sided colon. These drugs can be taken orally, rectally, or intravenously, and they can effectively control disease activity in the short term. However, due to its side effects, long-term use is not recommended. If stopping hormone drugs causes a relapse, doctors often recommend adding other drugs. Please note that these medications should not be abruptly discontinued. | Generally speaking, it is not recommended for IBD patients to use hormones for a long time. Hormones have powerful pharmacological effects such as anti-inflammatory, anti-allergic, immune suppression, and anti-tumor effects. The application of hormones for a certain period of time can achieve the goal of controlling clinical symptoms and reducing disease activity. The use of hormones may indicate that your disease is in an active inflammatory phase, and traditional medications such as mesalazine are ineffective. Hormones are generally used for a period of time and are not recommended for long-term use. Long-term use of hormones may cause side effects such as hypercortisolism, immunosuppression, anxiety and depression. | Due to the high incidence of adverse reactions and recurrence risk in hormone therapy for IBD, long-term use of hormone therapy is not recommended. Long-term use of hormones can easily lead to adverse reactions such as osteoporosis and pneumocystis pneumonia, and increase the risk of infection with pathogens. Although the use of hormones can alleviate symptoms in the short term, in the long run, when the dosage of hormones is gradually reduced, patients need to face the recurrence of the disease. In addition to hormones, IBD can choose other treatment options such as biological agents and immune modulators, which have clear efficacy and fewer side effects. |
| 2.Can immunosuppressants be used for a long time? | Immunosuppressants can be used as long-term maintenance therapy for IBD to help control inflammation and prevent recurrence. Common drugs include azathioprine, 6-mercaptopurine (6-MP), and methotrexate. These drugs reduce inflammation by suppressing the activity of the immune system, helping to maintain remission. Although immunosuppressants are generally safe when used for long periods of time, there may be some potential side effects and risks that need attention, including an increased risk of infection, liver toxicity, bone marrow suppression, and the risk of malignancies such as skin cancer and lymphoma. Therefore, during the medication process, it is necessary to conduct regular blood tests, maintain good hygiene habits, timely receive vaccinations such as influenza and pneumonia vaccines, avoid contact with infection sources, conduct regular skin examinations, make regular outpatient visits, discuss the condition and treatment plan with the doctor, and adjust the drug dose or change the medication in a timely manner. | IBD patients can use immunosuppressants for a long time, and these drugs are very effective in maintaining disease remission and reducing recurrence. Immunosuppressants, such as azathioprine, 6-mercaptopurine, and methotrexate, reduce inflammation by suppressing the overreaction of the immune system, thereby controlling the symptoms of IBD. Although long-term use of immunosuppressants can bring significant clinical benefits, potential side effects such as increased risk of infection, liver toxicity, and bone marrow suppression also need to be considered. Therefore, during the long-term treatment process, doctors will regularly monitor the patient's blood indicators and liver function to detect and deal with possible problems in a timely manner. | IBD patients can use immunosuppressants for a prolonged period, and these medications are highly effective in maintaining disease remission and reducing recurrence. Immunosuppressants such as azathioprine, 6-mercaptopurine, and methotrexate work by inhibiting the overreaction of the immune system to reduce inflammation, thereby controlling the symptoms of IBD. Although long-term use of immunosuppressants can provide significant clinical benefits, it is also essential to consider potential side effects such as an increased risk of infection, liver toxicity, and bone marrow suppression. Therefore, during the long-term treatment process, doctors will regularly monitor the patient's blood parameters and liver function to promptly identify and address any potential issues that may arise. | As one of the important therapeutic drugs for IBD, immunosuppressants can be used for a long time to maintain remission. However, patients need to take medication under the guidance of a doctor and strictly follow the medication guidelines to ensure medication safety. At the same time, patients also need to pay attention to strengthening personal protection and regularly testing blood indicators, including blood routine and liver and kidney function. | IBD is a chronic disease that usually requires long-term treatment to control the condition and prevent recurrence. Immunosuppressants, as maintenance therapy drugs, can help patients achieve this goal. It is very important to correctly choose effective drugs and their reasonable doses for treatment, in order to achieve disease remission and mucosal healing, and minimize the side effects of drugs. Immunosuppressants may have adverse effects on the body, such as decreased immune function and gastrointestinal discomfort. Therefore, patients need to be treated with medication under the guidance of a doctor to avoid adverse consequences caused by improper medication. In addition, patients need to pay attention to a light diet during treatment and avoid eating spicy and stimulating foods such as chili and pepper. | It can be used for a long time. Patients who have effective remission with azathioprine maintenance withdrawal of steroids, the course of immunosuppressant treatment is generally not less than 4 years. For IBD patients, immunosuppressants are commonly used to maintain remission, alleviate symptoms, and reduce the risk of disease recurrence. Long-term use of immunosuppressants may be part of the treatment of IBD, but this should be done under the supervision of a doctor, with a treatment plan tailored to individual circumstances. |
| 3. When can I be treated with biologics? | The timing of initiating biologic therapy for IBD patients should be based on several factors: if patients have poor response or cannot tolerate traditional treatments such as aminosalicylates, steroids, and immunosuppressants, biologic therapy may be considered. In severe or acute attacks, such as frequent diarrhea, severe abdominal pain, weight loss, etc., it may be necessary to start biological agent treatment as soon as possible to control symptoms and inflammation. If a patient develops complications related to IBD, such as fistula, abscess, or stenosis, biologics may help treat these complications. For some patients, especially those who show high-risk characteristics such as young onset, extensive intestinal involvement, and deep ulcers at the time of diagnosis, early use of biologics may help improve long-term prognosis. When considering treatment options, the patient's quality of life, personal preferences, and acceptance of treatment are also important factors. If a patient wishes to reduce steroid use or avoid long-term side effects, biologics may be an option. | IBD patients may consider starting biological therapy in the following situations: when patients have poor response or intolerance to traditional treatments such as aminosalicylates, steroids, and immunosuppressants, biological therapy may be considered. In severe or acute episodes, such as frequent diarrhea, severe abdominal pain, or significant weight loss, biologics may be necessary. If a patient develops complications related to IBD, such as fistulas, abscesses, or intestinal stenosis, biologics may help treat these conditions. For patients with high-risk characteristics at the time of diagnosis, such as young onset, extensive intestinal involvement, and deep ulcers, early use of biologics may help improve long-term prognosis. If a patient wishes to reduce the use of steroids or avoid their long-term side effects, biologics may be a suitable option. Based on the doctor's advice and the patient's specific condition and treatment response, it is decided whether to start biological agent therapy. | IBD patients may consider starting biologics therapy in the following situations: when patients have poor response or intolerance to traditional treatments such as aminosalicylates, steroids, and immunosuppressants, biologics may be an option. During acute attacks or severe conditions of the disease, such as frequent diarrhea, severe abdominal pain, or significant weight loss, biologics may help quickly control symptoms and inflammation. If a patient develops complications related to IBD, such as fistula, abscess, or stenosis, biologics can help treat these complications. For patients with high-risk characteristics at the time of diagnosis, such as young onset, extensive intestinal involvement, and deep ulcers, early use of biologics may improve long-term prognosis. When patients wish to reduce the use of steroids or avoid their long-term side effects, biologics may be a suitable option. The doctor will provide professional advice based on the patient's specific condition, symptoms, and treatment response to determine whether it is necessary to start biological agent therapy. | The decision to initiate biologic therapy requires an assessment of the severity of the IBD patient. For patients with moderate to severe IBD, including Crohn's disease and ulcerative colitis, if they do not respond well or cannot tolerate traditional treatments such as glucocorticoids and immunosuppressants, they should consider biological therapy. For example, if there are side effects during hormone therapy or the hormone cannot be completely stopped, biologics can be used to help reduce dependence on hormones. If a patient is preparing for colon resection surgery, biological therapy may help control disease activity and reduce surgical risks. In addition, for patients with high-risk factors for poor prognosis, early treatment with biological agents is also recommended. | The time to start using biological agents for treatment needs to be comprehensively evaluated and judged by doctors based on the specific circumstances of the patient, such as the presence of high-risk factors for the disease, the severity of the condition, the length of the medical history, and the response to drug treatment. Generally speaking, for patients with more serious conditions, such as severe intestinal inflammation, intestinal stenosis, intestinal obstruction, anal fistula, extraintestinal manifestations, and patients with a tendency towards cancer, we may recommend the use of biological agents. For patients with mild disease, if the drug treatment is not effective, biological agents can also be considered. For patients with high-risk factors such as young age, extensive involvement of the disease, and severe initial symptoms, early use of biological agents can also benefit them. It should be noted that biologics are not suitable for all IBD patients, and their use needs to strictly follow the doctor's advice and guidance. Before using biological agents, patients should fully understand their possible efficacy and side effects, and actively cooperate with the doctor's treatment plan. | The timing of starting biological therapy for IBD patients mainly depends on the severity and type of disease, the presence of high-risk factors, the therapeutic effect and tolerance, and the overall health status of the patient. Biologic therapy is usually targeted at patients with moderate to severe disease or those who have poor response to traditional treatment. If patients do not respond well or cannot tolerate traditional treatments such as 5-aminosalicylic acid drugs and hormones, biologics may be a suitable treatment option. For IBD patients with high-risk factors for poor prognosis, such as perianal lesions, extensive lesions, and concomitant esophagogastric and duodenal lesions, early treatment with biological agents is recommended. |
| 4. How do I choose the right biologic for me? | Choosing the right biological agent for oneself is typically a decision made after detailed discussion and assessment with one's IBD physician. Factors to consider include:   1. Disease Type: Ulcerative colitis and Crohn's disease differ pathologically and clinically, so certain biological agents may be more effective for one disease than the other. 2. Severity: The degree of disease activity and severity is an important factor in selecting a biological agent. For severe active disease, a more potent biological agent may be necessary to rapidly control inflammation. 3. Past Medication History: A patient's past response to other medications is an important consideration in selecting a biological agent. If there is no response or adverse reactions to certain drugs, other types of biological agents may be chosen. 4. Comorbidities: If a patient has comorbidities such as arthritis, skin lesions, or hepatobiliary diseases, a biological agent that also has therapeutic effects on these symptoms may be considered. 5. Safety: Biological agents can cause side effects such as infections, allergic reactions, and immune reactions. It is necessary for patients and doctors to jointly assess the risks and benefits of treatment and choose the most suitable biological agent for you. 6. Medication Regimen: The frequency of use and mode of administration of biological agents may also affect your choice. Some biological agents require weekly injections, while others may be infused every two months. 7. Health Insurance Coverage: When selecting a biological agent, it is also necessary to consider health insurance coverage and treatment costs. | When choosing the right biologic for IBD patients, the following factors should be considered: 1 Characteristics of the disease: Different biological agents may have different effects on different subtypes of IBD, such as ulcerative colitis or Crohn's disease. Therefore, it is important to first understand the type and severity of your condition, as well as your past response to other treatments. 2. Treatment history: Consider previous treatment plans and their effects. If you have used other biologics before and have not had significant results, you may need to try a different biologic. 3. Medical monitoring and support: Discuss and jointly develop treatment plans with doctors, including selecting appropriate biological agents. Doctors will provide personalized advice and support to patients based on their specific conditions and needs. 4. Safety and side effects: Understand the possible side effects and safety risks of biologics, as well as how to deal with possible adverse reactions. Different biological agents may have different safety risks, so it is necessary to consider the individual's health status and risk tolerance. 5. Convenience and treatment methods: Consider the administration methods and treatment frequency of biological agents, as well as their impact on lifestyle and daily life. Some biologics may require injections, while others may be administered through intravenous infusion. | When choosing the right biologic for IBD patients, there are several key factors to consider: 1 Type and severity of disease: understand your type of disease ulcerative colitis or Crohn's disease and the severity of your condition. Different biological agents may have different effects on different types of IBD. 2. Treatment history: Consider previous treatment plans and their effects. Knowing the reaction to previous biological agents, immunosuppressants, or other medications can help in selecting a new treatment plan. Safety and side effects: Learn about possible side effects and safety risks of biologics. Different biological agents may have different side effects, such as infection risk, allergic reactions, etc. It is necessary to consider the individual's health status and risk tolerance comprehensively. 4. Administration method and convenience: Consider the administration method of biological agents, such as subcutaneous injection, intravenous injection, and the frequency of treatment. Choosing a medication delivery method that suits your lifestyle and needs can help improve the convenience and compliance of treatment. 5. Medical monitoring and support: Work closely with doctors to develop personalized treatment plans. | When selecting biological agents for the treatment of IBD patients, it is necessary to consider multiple aspects such as disease assessment, drug characteristics, patient characteristics, drug safety and tolerability, treatment goals and expectations, and economic factors. First, the doctor needs to conduct a comprehensive assessment of the patient's IBD condition, including the severity of the condition, activity, extent of the lesion, and whether there are other complications. The mechanisms of action and indications of different biological agents vary, so it is necessary to choose the most suitable drug based on the specific situation of the patient. At the same time, it is necessary to consider factors such as the patient's age, gender, weight, comorbidities, allergy history, infection history, and immune status. When selecting biological agents, it is necessary to evaluate the patient's tolerance and safety. For elderly patients, patients with multiple diseases or poor physical function, more careful selection of biologics is needed. Finally, it is also necessary to consider factors such as the patient's treatment goals, expectations, and economic status. | When selecting a biologic agent suitable for IBD patients, it is necessary to consider multiple factors, including disease assessment, drug mechanism and target, individual differences and comorbidities, drug safety and tolerability, economic factors, and patient preferences. By comprehensively evaluating these factors, the most suitable biological treatment plan for the patient can be selected. You can make a comprehensive assessment and choice based on your condition, medical history, drug treatment response and other factors under the guidance of a doctor. Different biological agents are suitable for different types of IBD, as well as the severity of the disease. The efficacy and safety of biological agents are also important factors for patients to consider when choosing drugs. | The decision to use which biologic agent should be made based on the patient's condition, medication history, allergy history, and other factors. There are currently four types of commonly used biological agents: infliximab monoclonal antibody, adalimumab monoclonal antibody, vildagliptin monoclonal antibody, and ulinastatin monoclonal antibody. Common adverse drug reactions of biologics include allergic reactions, infections including tuberculosis, sepsis, congestive heart failure, infusion reactions, injection site reactions, malignancies, autoimmune reactions/autoantibodies, neurological adverse events, hematologic diseases, hepatitis B virus reactivation, and hepatobiliary adverse events. Since biological agents are antibody drugs, the issue of efficacy reduction due to drug immunogenicity also needs to be considered, which is another risk faced by patients. Only by facing up to these risks can we more likely achieve precise selection of different drugs. |
| 5. When do I need to undergo surgical treatment? | Surgical treatment is usually the last treatment option for IBD patients, and is only considered when other treatments fail to control the disease or severe complications occur, including ineffective medical treatment, intestinal obstruction, intestinal perforation, obstruction caused by intestinal stenosis, intestinal fistula, massive gastrointestinal bleeding, suspected cancerous changes, or severe complications related to intestinal diseases such as pericecal abscesses. These serious complications may require surgical treatment. | Patients with IBD usually need surgery in the following situations: when drug treatment is ineffective and the condition continues to worsen, surgery may become a necessary option. In addition, if the patient has serious complications such as intestinal obstruction caused by intestinal stenosis, intestinal perforation, massive bleeding, or the formation of fistulas and abscesses, surgery is often inevitable. For patients with ulcerative colitis, if there is a significant increase in the risk of precancerous lesions or colon cancer, surgery may be a preventive measure. The purpose of surgery can be to relieve symptoms, improve quality of life, or prevent further health problems. Each patient's condition is different, so the decision to undergo surgery should be based on a detailed evaluation by a doctor and a full understanding by the patient to ensure the most appropriate treatment plan is chosen. | Patients with IBD usually need to consider surgical treatment when drug treatment is ineffective or the condition continues to deteriorate. If severe complications arise, such as intestinal obstruction caused by intestinal stenosis, intestinal perforation, massive bleeding, or the formation of fistulas and abscesses, surgery is often inevitable. For patients with ulcerative colitis, if there is a significant increase in the risk of precancerous lesions or colon cancer, surgery may become a preventive measure. The purpose of surgery is to alleviate symptoms, improve quality of life, or prevent further health problems. Each patient's condition is different, and the decision to undergo surgery should be based on a detailed evaluation by the doctor and full understanding by the patient to ensure the most appropriate treatment plan is chosen. | When the chronic inflammation of IBD recurs frequently, leading to severe complications such as intestinal obstruction, internal/external fistulas, perforation, or carcinogenesis, surgery is often inevitable. When IBD patients have perianal diseases, such as perianal abscesses, and the presence of an abscess cavity is confirmed, emergency surgery is required to drain the pus, relieve pain, and prevent further progression of the disease. Additionally, surgery can be considered when medical treatment is ineffective or when the condition poses a serious threat to life and health. | The main situations in which IBD patients require surgical treatment are as follows. For patients with ulcerative colitis: if the condition cannot be controlled through comprehensive medical treatment and life-threatening complications arise, such as massive bleeding, intestinal perforation, or carcinogenesis, surgical treatment should be performed immediately. For complications that are not immediately life-threatening, such as refractory pancolitis or toxic megacolon, elective surgery can be considered. For patients with Crohn's disease: up to half of patients may require at least one surgical intervention. The surgical indications are similar to those for ulcerative colitis, including intestinal obstruction, fistula formation, abscess formation, acute intestinal perforation, and massive bleeding. | The indications for surgical resection in patients with ulcerative colitis and Crohn's disease are as follows. For patients with ulcerative colitis, the absolute indications include severe massive bleeding, perforation, carcinogenesis, and a high suspicion of carcinogenesis. Relative indications include severe ulcerative colitis that is unresponsive to aggressive medical treatment, particularly in cases with toxic megacolon, where early surgical intervention is recommended. Surgical intervention is also a viable option for patients who have poor response to medical treatment or whose quality of life is severely impacted by adverse drug reactions. For patients with Crohn's disease, indications for surgical intervention include intestinal obstruction, abdominal abscesses, fistula formation, acute perforation, massive bleeding, and carcinogenesis. Severe Crohn's disease patients who do not respond to medical treatment may require surgical intervention. Additionally, poor response to medical treatment or the occurrence of severe adverse drug reactions may also necessitate consideration of surgical intervention. |
| 6. What is Upadacitinib? | Uparitinib is an oral medication used to treat autoimmune diseases such as IBD. It reduces inflammation in the body by inhibiting an enzyme called Janus kinase 1 JAK1. Specifically, upatinib interferes with the JAK-STAT signaling pathway, which plays a key role in many immune responses. Inhibiting it can reduce the production of pro-inflammatory cytokines, thereby alleviating inflammation. Upatinib is a convenient oral medication that is taken once daily. It is typically used for patients with moderate to severe ulcerative colitis who have not responded to or cannot tolerate traditional treatment methods. When using upatilumab, the doctor will decide whether it is suitable for you based on your specific condition and treatment history, and will regularly monitor the therapeutic effect and side effects. Common side effects include upper respiratory tract infections, headaches, gastrointestinal discomfort such as nausea and diarrhea, and hypertension. | Uparatinib is an oral small molecule drug belonging to the JAK inhibitor class, which is used to treat moderate to severe active ulcerative colitis and other inflammatory diseases. JAK inhibitors reduce inflammatory responses in the body by inhibiting the Janus kinase JAK pathway, which is very effective in regulating the overactive state of the immune system. Uparitide can block the signaling of multiple cytokines, thereby reducing the activity of inflammatory cells and related symptoms. Uparitide is commonly used in patients who have failed or are intolerant to traditional treatments such as immunosuppressants and biologics. It can significantly alleviate the symptoms of ulcerative colitis, such as abdominal pain, diarrhea, and intestinal bleeding, and help improve the overall quality of life of patients. Uparitini should be used under the guidance of a doctor during the treatment process, and regular monitoring is needed to evaluate the efficacy and monitor possible side effects, such as infection risk and abnormal blood indicators. | Upadacitinib is an oral JAK inhibitor specifically used to treat moderate to severe active ulcerative colitis and other autoimmune and inflammatory diseases. Its mechanism of action is to inhibit specific members of the Janus kinase JAK family, interfere with intracellular signaling pathways, and thereby reduce inflammatory responses. This medication effectively alleviates symptoms of IBD patients, such as abdominal pain, diarrhea, and rectal bleeding, and helps promote intestinal healing. Uparitide is usually used for patients who have poor response or cannot tolerate traditional treatment methods such as immunosuppressants and biologics. Its use requires strict monitoring by a doctor, as it may cause some side effects, such as increased risk of infection, hematological abnormalities, and liver function damage. The efficacy and safety of upatilimab have been verified in multiple clinical trials, showing its significant advantages in long-term symptom control and improving patients' quality of life. | Uparatinib is an oral targeted synthetic small molecule drug belonging to the JAK inhibitor class, used to treat certain immune-mediated inflammatory diseases. Uparatinib interferes with a series of cell signaling pathways related to inflammation and immune response by inhibiting the activity of JAK enzymes. This can reduce the production and release of inflammatory mediators, thereby alleviating inflammatory symptoms and tissue damage. Upatec is approved for moderate to severe active rheumatoid arthritis, active psoriatic arthritis. In 2023, Upadacitinib was also approved by China's CFDA for the treatment of moderate to severe active ulcerative colitis and moderate to severe active Crohn's disease that have poor response to one or more TNF inhibitors, or are intolerant or contraindicated. As an oral medication, it is convenient to use and is more acceptable to patients than injectable drugs. The most common side effects of upatilimab are herpes zoster and lymphopenia; Other conditions such as severe infections and a possible increased risk of malignancy may also occur. | Uparatinib is an oral selective JAK1 inhibitor with a clear mechanism of action and clinical application for the treatment of various immune diseases. By inhibiting the activity of JAK-1 and blocking the JAK-mediated signaling pathway, it has a regulatory effect on the immune system, thereby exerting a significant therapeutic effect on inflammation. Uparitide is mainly used to treat moderate to severe rheumatoid arthritis, moderate to severe active ulcerative colitis, moderate to severe active Crohn's disease, etc. that have not responded to first-line treatment. Upatinib may cause some side effects, such as upper respiratory tract infection, nausea, headache, and hypertension. In rare cases, there may be serious risks of infection, thrombosis, and abnormal liver function. Therefore, regular monitoring and evaluation are required during use. | Uparitide is an oral medication used to treat autoimmune diseases such as IBD. It reduces inflammation in the body by inhibiting an enzyme called JAK1. Uparitinib is a convenient oral medication that is taken once daily. It is commonly used for patients with moderate to severe ulcerative colitis and Crohn's disease who have failed or are intolerant to traditional treatment methods. Compared with non-enteral administration of biologics, upatilimab is administered orally, eliminating the adverse reactions associated with intravenous or subcutaneous injection. In addition, upatilimab has low immunogenicity, short serum half-life, and high intestinal bioavailability. When using upatilumab, it is necessary to determine whether it is suitable for use based on the specific condition and treatment history of the patient, and to regularly monitor the therapeutic effect and side effects. Common side effects include upper respiratory tract infection, headache, gastrointestinal discomfort such as nausea and diarrhea, and hypertension. |
| 7. When do I need enteral nutrition? | Parenteral nutrition is an important nutritional support method in the management of IBD, and is usually used for patients who cannot receive sufficient nutrition through oral or enteral nutrition. Whether to require parenteral nutrition should be comprehensively evaluated and decided by doctors based on specific circumstances. For patients with IBD, parenteral nutrition may be considered in the following situations: 1 Severe malnutrition: If a patient is severely malnourished due to illness and cannot receive adequate nutrition through oral or enteral nutrition, parenteral nutrition may be necessary. This may happen in patients with severe Crohn's disease or ulcerative colitis. 2. Intestinal dysfunction: In some cases, the patient's intestines may not be able to absorb nutrients normally, such as intestinal obstruction, extensive small intestinal lesions, or intestinal fistulas. These conditions can lead to malabsorption of nutrients and require supplementation through parenteral nutrition. 3. Postoperative recovery: After certain intestinal surgeries, especially those involving extensive resection or reconstruction, patients may require short-term or long-term parenteral nutrition until intestinal function is restored. 4. Severe acute attack: In the severe acute attack period of IBD, if oral or enteral nutrition will aggravate the symptoms or cause complications, parenteral nutrition can be used as a temporary nutritional support. 5. Treatment of complications: Some complications, such as severe intestinal inflammation, abscesses, or extensive intestinal ulcers, may require nutritional support through parenteral nutrition while allowing the intestine to rest and recover. 6. Preparation or replacement surgery: In some cases, parenteral nutrition can be used for preoperative preparation, especially in patients with poor nutritional status, to improve surgical tolerance and postoperative recovery. 7. No enteral nutrition route: In rare cases, when all enteral nutrition routes are not feasible, such as extensive short bowel syndrome, patients may need to rely on parenteral nutrition for a long time. | Parenteral nutrition is usually considered for patients with IBD in the following situations:1 Malnutrition: If a patient has insufficient nutrient intake due to disease activity, intestinal absorption disorders, or long-term diarrhea, and cannot take enough nutrients orally, parenteral nutrition may be necessary. In this case, parenteral nutrition can provide the nutrients needed by patients, maintain body weight, promote health, and avoid malnutrition. 2. Bowel rest: In severe cases of IBD, sometimes doctors recommend temporarily stopping oral intake to allow the bowel to fully rest and heal. In this case, parenteral nutrition can be used as a temporary substitute to meet the nutritional needs of patients while reducing the burden on the intestine. 3. Intestinal obstruction or fistula formation: If a patient experiences intestinal stenosis, obstruction, or fistula, oral intake of food may cause further complications. At this time, parenteral nutrition can bypass the affected intestinal area and provide direct nutritional support, avoiding the passage of food through the affected area. Before and after surgery: Before and after IBD-related intestinal surgery, especially surgery involving intestinal resection, parenteral nutrition can help patients prepare for surgery and promote postoperative recovery. | Patients with IBD usually need parenteral nutrition in the following situations: 1 Severe malnutrition: When patients are unable to take in enough nutrients through the mouth or intestines due to severe abdominal pain, diarrhea, nausea, or vomiting, parenteral nutrition can provide essential nutritional support. Malnutrition can weaken the immune system and delay the healing process, so timely supplementation of nutrients is crucial. 2. Intestinal dysfunction: Some patients with IBD may experience severe intestinal dysfunction, such as extensive intestinal stenosis, fistula, abscess, or widespread inflammation, making it difficult for food to pass through the digestive tract normally. At this time, enteral nutrition may not be able to meet the body's needs, and parenteral nutrition becomes a necessary alternative. 3. Post-operative recovery: In some cases where surgery is required, such as after bowel resection, the intestine needs time to heal and restore function. Parenteral nutrition may be needed to maintain the nutritional status of the body in the early stages after surgery until the intestinal function returns to normal and the body can resume absorbing nutrients through the intestine. 4. Intestinal obstruction: If a patient has intestinal obstruction due to inflammation or other complications, food cannot pass through the intestines, and parenteral nutrition can temporarily resolve the obstruction problem while providing necessary nutritional support. Complex fistulas and abscesses: For patients with complex fistulas or abscesses, especially those requiring surgery, parenteral nutrition can help reduce the burden on the intestine and provide the necessary nutrients to support the body's healing process. 6. Inability to tolerate enteral nutrition: In some cases, patients may be unable to ingest sufficient nutrients through the enteral route due to severe gastrointestinal reactions or intolerance to enteral nutrition. In such cases, parenteral nutrition is an important alternative. | IBD patients may need enteral nutrition support in cases of insufficient nutrient intake, limited effect of drug treatment, and imbalance of water and electrolytes. When patients are unable to take food orally or have insufficient food intake due to disease causes such as abdominal pain, diarrhea, and loss of appetite, or even malnutrition, enteral nutrition support should be considered to improve nutritional status and enhance the patient's resistance. When medication is effective but the patient's weight continues to decrease, enteral nutrition can be used as an adjuvant treatment to help patients regain weight and nutritional status. In addition, after intestinal surgery, during severe acute attacks of IBD, or when serious complications occur, enteral nutrition plays an important role in alleviating the patient's condition. | For IBD patients, enteral nutrition can effectively improve their nutritional status, enhance their resistance, and promote the recovery of the disease. When the disease causes long-term diarrhea, abdominal pain, and loss of appetite, resulting in insufficient nutrient intake and weight loss, such as a weight loss of ≥5% in the past 3 to 6 months and a BMI below the normal range of 18.5kg/m², enteral nutrition support should be considered. For patients with severe diarrhea, enteral nutrition can not only provide essential nutrients, but also help replenish the water and electrolytes lost due to diarrhea, maintaining the patient's water and electrolyte balance. Secondly, when the patient's nutritional status continues to deteriorate and their weight continues to decrease despite medication, enteral nutrition can be used as an adjuvant treatment to prevent malnutrition. In addition, for patients with Crohn's disease, enteral nutrition can help alleviate the disease. | The role of nutritional support therapy for ulcerative colitis is mainly to improve malnutrition and prevent nutritional risks, while its more important role for Crohn's disease is to induce and maintain remission of Crohn's disease. It is recommended that IBD patients undergo regular screening, especially perioperative patients, to identify individuals at risk of malnutrition or malnutrition. When the surgical indications are clear and the patient has nutritional problems or potential risks, it is strongly recommended to provide nutritional support therapy before surgery to reduce the risk of surgery. For children and adolescents with active Crohn's disease, total enteral nutrition is the first-line option for inducing remission, which is as effective as steroids. The preferred method of inducing remission is the use of total enteral nutrition. For adult patients with Crohn's disease, although the effect of enteral nutrition induction of remission is not as good as that of hormones, enteral nutrition can be considered as an alternative treatment for inducing remission in cases where drug therapy is ineffective or contraindicated, and can be used as an effective nutritional support and adjuvant treatment regimen. |
| 8. Is fecal transplantation useful? | Fecal microbiota transplantation is a treatment method that transfers the fecal microbiota of healthy donors to the intestines of patients. It has been shown to be potentially effective in treating certain intestinal diseases, especially Clostridium difficile infection. For IBD patients, the effect and application of fecal microbiota transplantation are still under study, but some preliminary studies have shown potential benefits. IBD patients usually have an imbalance of intestinal flora. Fecal microbiota transplantation can help restore a healthy balance of bacteria, thereby reducing inflammatory responses. Some studies have shown that fecal microbiota transplantation may help alleviate symptoms of IBD patients, such as abdominal pain, diarrhea, and intestinal bleeding. In addition, some clinical trials have shown that fecal microbiota transplantation can induce disease remission in some IBD patients, especially in cases where other treatments have failed. | Fecal microbiota transplantation is an emerging treatment for IBD that is being studied and has shown potential benefits in some cases. Research shows that the microbial composition of the human gut, known as the gut microbiota, is closely related to the occurrence and development of IBD. Fecal microbiota transplantation aims to restore or improve the intestinal microecological balance of patients by transplanting the microbial community in the feces of healthy donors into the intestines of patients. Although the current research is still in its early stages, some findings suggest that fecal microbiota transplantation may have some degree of benefit for certain IBD patients, especially for potentially refractory infections or difficult-to-control cases. Some clinical trials have shown that fecal microbiota transplantation can alleviate the symptoms of IBD, improve intestinal function, and even promote remission of the disease. However, there are still some challenges and risks in fecal microbiota transplantation, including the spread of infectious diseases, the imbalance of intestinal microecology, and potential adverse reactions. Therefore, fecal microbiota transplantation is usually reserved for clinical trials and severe IBD patients in specific situations, and needs to be conducted under the guidance of a doctor. | Fecal microbiota transplantation is a treatment method that restores the balance of intestinal flora by transplanting the microbial community in the feces of healthy people into the intestines of patients. For the treatment of IBD, the efficacy of fecal microbiota transplantation is still under study, and there are some limitations and risks. Current research shows that fecal microbiota transplantation may have some potential for the treatment of IBD, especially for ulcerative colitis and some patients with Crohn's disease. Fecal microbiota transplantation can regulate the immune system by improving the diversity and balance of intestinal flora, reducing inflammatory reactions, and thus improving symptoms. However, further research is needed to elucidate the precise mechanism and therapeutic efficacy of fecal microbiota transplantation. Although fecal microbiota transplantation has certain potential, there are also some limitations and risks. Firstly, the long-term effects of fecal microbiota transplantation are still unclear, and more clinical studies are needed to verify its safety and effectiveness. Secondly, fecal microbiota transplantation may cause infection, allergic reactions, or other adverse reactions. In addition, the operation of fecal microbiota transplantation requires professional equipment and operational techniques, as well as ensuring the safety and reliability of the donor. | Fecal transplantation involves introducing functional bacteria from healthy individuals into the patient's intestine to reconstruct missing gut flora, adjust microecological balance, and treat both internal and external intestinal diseases. After undergoing fecal transplantation, IBD patients must pay attention to dietary hygiene and avoid eating spoiled or unclean foods, especially raw or cold foods, to prevent digestive tract infections. After transplantation, patients also need to avoid strenuous exercise to prevent affecting the peristalsis of the gastrointestinal tract and affecting the absorption of nutrients. Due to different physical conditions and different surgical methods, rejection reactions may occur after surgery. Therefore, it is necessary to take immunosuppressants under the guidance of a doctor and regularly visit the hospital for follow-up examinations to avoid complications after surgery. | Fecal transplantation has shown certain efficacy in IBD patients, capable of improving disease symptoms and alleviating disease activity to a certain extent. However, when applying fecal transplantation as a treatment, doctors need to comprehensively consider the patient's specific conditions and potential risks to ensure the safety and effectiveness of the treatment. Fecal transplantation can alter the intestinal microenvironment of IBD patients and increase intestinal stability by enhancing the diversity of fecal bacteria. Nevertheless, in general, fecal transplantation cannot achieve a complete cure for IBD but can typically only alleviate clinical symptoms. Therefore, patients also need to adopt other treatment methods according to their doctor's advice to control the condition. | Fecal transplantation may have therapeutic effects on IBD patients. Clinical trials have shown that fecal microbiota transplantation can induce disease remission in some IBD patients, especially in cases where other treatments have failed. After fecal transplantation treatment, 63% of IBD patients experienced clinical remission, and 76% of patients could stop using IBD-related treatment drugs and improve their clinical symptoms. Since 2013, many countries' guidelines, including China, the United Kingdom, the United States, and Europe, have included fecal microbiota transplantation in their treatment guidelines. Although fecal microbiota transplantation has shown some efficacy in the treatment of IBD, its application data in this patient population is still limited. Therefore, when deciding whether to use fecal microbiota transplantation for treatment, doctors need to consider the specific situation and potential risks of the patient. |
| 9. Can IBD be cured? | At present, IBD cannot be completely cured. However, with appropriate treatment and management, many patients can achieve long-term remission and a good quality of life. The main goals of IBD treatment are to control inflammation, relieve symptoms, maintain remission through long-term treatment, prevent recurrence, and reduce and prevent complications such as intestinal stenosis, fistula, and malnutrition. Scientists are constantly researching new treatments and drugs in order to find more effective treatment methods and potential cures. including stem cell therapy, gene therapy and new biological agents are all under research. | IBD is not yet completely curable. It is a chronic disease that requires long-term management and treatment. The main goal of treatment is to control symptoms, reduce inflammation, promote intestinal healing, and prevent complications. Through medication, lifestyle adjustments, and sometimes surgical intervention, many patients can achieve long-term remission and live a near-normal life. Nonetheless, IBD typically exhibits recurring episodes, necessitating ongoing medical monitoring and management. | IBD is currently incurable but can be effectively managed and controlled through various treatment methods. IBD encompasses ulcerative colitis and Crohn's disease, both of which are chronic and recurrent, typically requiring long-term treatment and monitoring. The primary goals of treatment are to alleviate symptoms, maintain remission, prevent complications, and improve quality of life. Common treatment approaches include pharmacotherapy, nutritional support, and surgical intervention. Pharmacological treatment may encompass anti-inflammatory drugs, immunosuppressants, and biologics, which can help control inflammation, reduce symptoms, and prolong remission periods. While a cure is not available, many patients can achieve long-term symptom relief and lead a normal life through appropriate treatment and lifestyle adjustments. However, disease management necessitates individualized treatment plans, along with regular follow-ups and adjustments to treatment strategies to address changes and progressions in the disease. | IBD is currently incurable. It is caused by abnormal functioning of the autoimmune system, leading to chronic damage to the intestinal mucosa. In treatment, salicylates or immunosuppressants, among others, are used to inhibit the immune inflammatory response of the intestinal mucosa and improve intestinal discomfort symptoms. As the abnormal functioning of the patient's immune system cannot be completely corrected, such patients may experience exacerbation of immune dysfunction and recurrence of intestinal discomfort symptoms under conditions such as poor emotional state or unhealthy dietary habits. Nevertheless, through appropriate treatment and management, many patients can achieve long-term remission and a good quality of life, achieving a state of clinical cure. | IBD is a chronic disease, and its pathological mechanisms involve multiple complex factors, including genetic, environmental, and immune system abnormalities. These factors work together on intestinal tissue, leading to long-term inflammatory reactions and damage, which are often irreversible. Although drug therapy can control symptoms, it cannot change the tissue structure of the lesion, so it cannot achieve radical cure. In some individuals, due to mild disease or timely and effective medical intervention, the disease may be in remission or even achieve clinical cure. Patients should undergo regular check-ups to monitor the progression of their condition. They should also follow medical advice to adjust their dietary habits and lifestyle, such as avoiding high-fat foods and maintaining adequate sleep, which can help alleviate symptoms and improve quality of life. | IBD is a chronic disease that cannot be fully cured at present, but its progression can be controlled through medication and adjustment of healthy lifestyle habits. Patients need to undergo treatment under the guidance of a physician while maintaining a positive mindset and attitude towards life. For patients with ulcerative colitis, active treatment can often lead to remission, especially in mild cases, enabling them to resume normal work and study without significant impairment. However, a minority of ulcerative colitis patients may experience acute onset, severe symptoms, or be of advanced age (over 60 years old), typically resulting in a poorer prognosis and a greater difficulty in achieving cure. Crohn's disease, a chronic inflammatory granulomatous disease of the digestive tract, is generally considered more severe than ulcerative colitis. Although mild cases may respond well to treatment, they are prone to recurrent episodes and prolonged illness. Additionally, some patients may experience severe complications such as intestinal obstruction or gastrointestinal perforation, leading to a poorer prognosis and difficulty in achieving cure. |
| 10. What is the ultimate therapeutic goal for IBD? | The ultimate goal of IBD treatment is to achieve and maintain long-term clinical remission, which means reducing or eliminating symptoms and enabling patients to lead a normal life. This includes the following specific objectives: 1 Control symptoms: alleviate symptoms such as abdominal pain, diarrhea, and bleeding, and improve the quality of life of patients. 2. Healing the intestinal mucosa: Reducing inflammation and damage to the intestine through treatment, promoting intestinal healing. 3. Prevent complications: Reduce the incidence of complications such as intestinal stenosis, perforation, abscess, fistula, etc. 4. Reduce the need for surgery: Reduce the risk of patients needing surgical treatment through effective drug therapy. 5. Maintain long-term remission: Maintain the stability of the disease during remission and prevent recurrence. 6. Improve the quality of life: Help patients return to normal life, work, and social activities. | The ultimate treatment goals of IBD are to achieve and maintain clinical remission, prevent complications, and enhance patients' quality of life. These objectives encompass the following aspects:   1. Symptom Control: To reduce or eliminate symptoms such as abdominal pain, diarrhea, and bleeding through treatment, enabling patients to lead normal lives and work routines. 2. Maintaining Long-Term Remission: It involves not only alleviating symptoms during acute episodes but also preventing disease recurrence through long-term treatment and monitoring. 3. Preventing Complications: This entails preventing both intestinal complications (e.g., strictures, fistulas, abscesses) and extraintestinal complications (e.g., arthritis, skin problems, ocular inflammation). 4. Promoting Intestinal Healing: Utilizing medications and other therapeutic methods to facilitate the healing of intestinal mucosa, thereby reducing inflammation and ulcer formation. 5. Enhancing Quality of Life: Assisting patients in managing the psychological and emotional stress associated with the disease, improving nutritional status, and bolstering overall health and well-being. | The ultimate treatment goals of IBD are to achieve and maintain disease remission while enhancing patients' quality of life. The specific objectives include:   1. Symptom Relief: Controlling abdominal pain, diarrhea, fatigue, and other symptoms through effective treatment to enable patients to lead normal lives. 2. Reducing Inflammation: Employing medications and other therapeutic interventions to diminish intestinal inflammation, thereby preventing further tissue damage and the onset of complications. 3. Promoting Healing: Facilitating the healing of intestinal mucosa to reduce the formation of ulcers and other lesions. 4. Preventing Recurrence: Maximizing the duration of remission and preventing disease recurrence through long-term maintenance therapy. 5. Preventing Complications: Monitoring and managing potential complications such as intestinal strictures, fistulas, malnutrition, and increased risk of colorectal cancer. 6. Improving Quality of Life: Assisting patients in maintaining normal living, work, and social activities through appropriate medical care and support, minimizing the impact of the disease on their daily lives. | The ultimate treatment goals of IBD are to induce and maintain clinical remission and mucosal healing through a comprehensive treatment strategy, prevent and control complications, and enhance patients' overall quality of life. These specifically encompass:   1. Minimizing or eliminating IBD symptoms to achieve and sustain an asymptomatic state (remission) for patients. 2. Intestinal Mucosal Healing: Promoting and maintaining complete healing of the intestinal mucosa, reducing inflammation and ulceration, thereby lowering the risk of disease recurrence and complications. 3. Preventing Complications: Effectively managing and preventing IBD-related complications such as strictures, fistulas, perforations, as well as extraintestinal manifestations like arthritis, skin, and eye problems. 4. Reducing the impact on daily life, work, and social activities by controlling the disease, thereby improving patients' overall quality of life and mental health. 5. Minimizing or mitigating the side effects of long-term medication use (e.g., steroids and immunosuppressants) during the treatment process. 6. Avoiding or delaying the need for surgical intervention through effective pharmacological treatment and disease management. | The treatment goals of IBD are multifaceted, aiming to control symptoms, promote mucosal healing, prevent complications, improve patients' quality of life, and prevent disease recurrence. Treatment necessitates the selection of appropriate therapeutic drugs and regimens based on the individual patient's specific condition and the location of the lesion.  The primary goal is to control IBD symptoms such as abdominal pain, diarrhea, and bloody stools, enabling patients to experience a marked improvement in their condition. During periods of active disease, pharmacological and other therapeutic interventions are employed to induce remission, allowing patients to return to a normal or near-normal life.  Secondly, IBD often leads to damage to the intestinal mucosa, making the promotion of mucosal healing and the reduction of persistent inflammatory damage to the intestinal mucosa one of the essential treatment objectives.  Thirdly, IBD can give rise to a range of complications, including intestinal obstruction, perforation, and massive bleeding. Treatment is essential to prevent these complications from occurring, ensuring patients' safety and well-being.  Lastly, in addition to controlling symptoms and preventing complications, treatment must also focus on patients' overall quality of life, encompassing psychological, social, and economic aspects. | The treatment goals of IBD encompass utilizing comprehensive therapeutic approaches to control the disease, alleviate symptoms, promote mucosal healing, prevent complications, and enhance patients' quality of life. The primary objective is to reduce or eliminate IBD symptoms, such as abdominal pain, diarrhea, and bloody stools, enabling patients to achieve a state of clinical remission. This necessitates physicians adopting suitable pharmacotherapeutic regimens tailored to individual patient circumstances to suppress inflammatory responses.  Secondly, it is crucial to promote intestinal mucosal healing in IBD patients to alleviate clinical symptoms, prevent the onset of related complications (e.g., intestinal obstruction, perforation), thereby improving quality of life and preventing disease recurrence.  To achieve these treatment objectives, concerted efforts from both physicians and patients are essential in devising personalized treatment plans and conducting regular follow-ups to monitor disease progression. |
| **Daily management** | | | | | | |
| 1. What is the best diet for IBD? | There is no one-size-fits-all "best diet" for IBD patients, as everyone responds differently to food. First, maintain a balanced diet and ensure the intake of various nutrients, including protein, carbohydrates, healthy fats, vitamins, and minerals. If tolerated, high-fiber foods such as fruits, vegetables, and whole grains can promote gut health, but may need to be reduced during severe symptoms. Secondly, avoid foods that may cause symptoms, such as high-fat foods, dairy products, and high-sugar foods; Avoid stimulating foods such as caffeine, alcohol, chili peppers, and pepper. Finally, try to eat small meals, maintain adequate water intake, and supplement specific vitamins and minerals. There is no one-size-fits-all "best diet" for IBD patients, as everyone responds differently to food. First, maintain a balanced diet and ensure the intake of various nutrients, including protein, carbohydrates, healthy fats, vitamins, and minerals. If tolerated, high-fiber foods such as fruits, vegetables, and whole grains can promote gut health, but may need to be reduced during severe symptoms. Secondly, avoid foods that may cause symptoms, such as high-fat foods, dairy products, and high-sugar foods; Avoid stimulating foods such as caffeine, alcohol, chili peppers, and pepper. Finally, try to eat small meals frequently, maintain adequate water intake, and supplement specific vitamins and minerals. | The optimal diet for IBD patients varies from person to person, but in general, it should be balanced, easily digestible, and nutritious. Patients need to pay attention to avoiding foods in their diet that may worsen symptoms, such as high fiber, greasy, spicy, or dairy products. A low residue diet is generally beneficial to the gut because it reduces the amount of fiber that is not easily digested, thereby reducing the burden on the gut. Patients should consume more protein-rich foods such as lean meat, fish, and soy products to promote healing and maintain weight, while also supplementing with sufficient vitamins and minerals to prevent malnutrition. For some patients, the serving of individual dishes and the method of eating smaller portions more frequently can help alleviate abdominal discomfort and reduce gastrointestinal stress. Because each patient's condition and physical response are different, it is recommended to develop a personalized diet plan under the guidance of a professional nutritionist or doctor, and adjust it according to changes in the patient's condition. Keeping a good diet record and monitoring the relationship between diet and symptoms can also help find the most suitable diet pattern for you. | The optimal diet for IBD patients should be individualized and adjusted according to the patient's symptoms and condition. Generally, the diet should be rich in nutrients and easy to digest, avoiding irritating the intestines. During remission, patients can choose fiber-rich foods such as whole grains, fruits, and vegetables to promote gut health. However, during the active phase of the disease, it may be necessary to reduce fiber intake to reduce intestinal burden and discomfort. In addition, a low-fat, low-residue, and low-lactose diet may be beneficial for some patients. Protein can be obtained from lean meat, fish, eggs and soy products to ensure adequate nutrient intake. Maintain hydration and avoid stimulants such as caffeine and alcohol. Documenting and monitoring food intake and its effect on symptoms can help identify and avoid personal triggers. In any case, it is best to develop and adjust a diet plan under the guidance of a nutritionist or doctor to ensure safe and effective diet. | For IBD patients, the best diet plan should be tailored to the patient's specific situation, nutritional needs, and disease stage. IBD patients should generally eat a variety of foods, pay attention to eating small amounts of food at regular intervals, and avoid eating raw, spicy, or overheated foods to prevent aggravating their condition. For patients with IBD in remission, there are several aspects to consider: first, a low-residue diet is recommended, and second, supplementation of trace elements is important to alleviate the symptoms of IBD. Patients in the acute phase should have a low-residue, low-fiber, liquid or semi-liquid diet, and try to choose foods that are easy to digest, avoiding or limiting the intake of non-water-soluble fibers. | The optimal diet for IBD needs to be adjusted according to the specific condition of the patient. Generally speaking, patients in the acute phase need to choose low-fat liquid or low-residue semi-liquid diets to avoid aggravating the burden on the intestines. For example, you can choose rice soup, steamed egg white, lotus root powder, vegetable juice and other dishes. During the remission period, patients can appropriately increase the variety and intake of food, but still need to pay attention to the texture and nutrition of the diet. In addition, patients should also pay attention to avoiding excessive consumption of certain foods that may aggravate the condition, such as high-fiber foods such as whole wheat bread, oatmeal, beans, spicy foods, caffeine, and alcohol. At the same time, if there are gastrointestinal symptoms or obvious stenosis, patients should reduce the intake of insoluble dietary fiber. For the treatment of IBD patients, oral nutritional preparations can be appropriately added when necessary to ensure the nutritional needs of patients. | The optimal diet for IBD patients should take into account the severity, stage and type of disease as well as the nutritional needs of the patient. During acute attack or perioperative period, low-fat liquid food or low-residue low-fat semi-liquid food is recommended, such as rice soup, lotus root starch, rice noodles, vegetable juice, steamed egg white, etc., while avoiding high-fiber foods and dairy products. During the remission period, it is possible to gradually increase the intake of low-fiber, low-residue foods, such as lean meat, fish, poultry, eggs, and other high-quality proteins, as well as fresh vegetables and fruits, especially dark-colored vegetables, to supplement vitamins and minerals. In addition, the diet should be low in fat, with fat intake limited to 50g-70g/d, and avoid fried and spicy foods. For patients with lactose intolerance, they should eliminate lactose-containing foods or choose sugar-free and lactose-free dairy products. In short, the diet of IBD patients should be based on easy digestion, low fiber, low fat, and sufficient protein, while ensuring the intake of vitamins and minerals to promote intestinal health. At the same time, dietary recommendations should be individualized and adjusted as the disease changes. |
| 2. What kind of foods should I avoid? | IBD patients should avoid the following types of foods, which may trigger or worsen symptoms:1 High-fat foods: Fried foods and high-fat meats may aggravate diarrhea and abdominal pain. 2. Dairy products: If there is lactose intolerance, dairy products such as milk, cheese, and ice cream may cause bloating, abdominal pain, and diarrhea. 3. High-sugar foods: Candy, cake, soda and other high-sugar foods may cause diarrhea and increased gas. 4. High-fiber foods: Raw vegetables, whole grains, nuts, and seeds may irritate the intestines and cause discomfort during exacerbation of symptoms. 5. Spicy and stimulating foods: Spicy foods such as chili peppers and black pepper may stimulate the intestines, causing abdominal pain and diarrhea. Caffeine and alcohol: Coffee, tea, carbonated drinks and alcohol may irritate the gut and increase the risk of diarrhea and dehydration. 7. Carbonated drinks: Sodas and sparkling waters may cause bloating and discomfort. 8. Artificial sweeteners: Artificial sweeteners such as sorbitol and xylitol are commonly found in sugar-free gum and some processed foods, which may cause diarrhea and flatulence. | IBD patients need to pay special attention to their diet, as certain foods may aggravate symptoms or trigger recurrence. Although specific dietary recommendations vary depending on individual circumstances, in general, the following foods should be avoided: 1 High-fiber foods: including raw fruits and vegetables, whole grains, and nuts. These foods may irritate the intestine, especially during active disease. 2. High-fat foods: such as fried foods, cream, fatty meat, and high-fat dairy products. A high-fat diet may worsen diarrhea and other digestive problems. 3. Dairy products: Some IBD patients may be intolerant to lactose, and dairy products can cause bloating, abdominal pain, and diarrhea. If this happens, avoid milk, cheese, and other dairy products. 4. High-sugar foods: including desserts, candies, and sugary drinks. High-sugar foods may cause diarrhea and gastrointestinal discomfort. Caffeine and alcohol: Coffee, tea, chocolate, and alcoholic beverages may irritate the gut and trigger worsening of symptoms. 6. Spicy and stimulating foods: such as chili, chili sauce and foods containing a lot of spices, which may aggravate abdominal pain and diarrhea. 7. Carbonated drinks: Sodas and other carbonated drinks may cause flatulence and discomfort. 8. Peels and seeds of high-fiber fruits and vegetables: Peels and seeds may be difficult to digest, especially during active disease, so it is best to eat them peeled or seeded. | IBD patients need to pay special attention to their diet, as certain foods may exacerbate symptoms or trigger a recurrence of the disease. Although everyone reacts differently to food, it is generally recommended to avoid the following types of food: High-fiber foods: especially rough fiber foods such as raw vegetables and fruits, whole grains, and nuts. These foods may irritate the intestines during acute attacks, causing abdominal pain and diarrhea. 2. High-fat foods: including fried foods, fatty meat, cream, and full-fat dairy products. High-fat foods may aggravate diarrhea and intestinal discomfort. Dairy products: Many people with IBD may be intolerant to lactose, which can cause bloating, abdominal pain, and diarrhea. Dairy products should be reduced or avoided unless it has been confirmed that you can tolerate them. 4. Spicy foods: Chili peppers, hot sauce, and other spicy condiments may irritate the intestines and worsen symptoms. 5. Caffeinated beverages: The caffeine in coffee, tea, and certain soft drinks may increase bowel movements, leading to diarrhea and discomfort. 6. Alcohol: Alcohol may irritate the gastrointestinal tract and exacerbate symptoms such as diarrhea and abdominal pain. 7. High-sugar foods, candies, cakes, cookies, and other high-sugar foods may increase diarrhea and intestinal gas. 8. Carbonated drinks: Carbonated drinks may cause bloating and gas accumulation, increasing discomfort. 9. Processed foods: Processed foods containing artificial additives, preservatives, and excessive salt may irritate the intestines. | For patients with IBD, including ulcerative colitis and Crohn's disease, regardless of whether they are in active or remission phase, they should eat in moderation. The following are specific suggestions: Rice and flour staple food: reduce pasta during the active phase, and adjust according to the condition of the stool during the remission phase. Vegetables: Eat less during active periods, and those at risk of stenosis/perforation should avoid eating; During remission, consume as appropriate. Fruits: Avoid raw fruits and eat less during the active stage; During the remission period, consume the apple in moderation, removing the skin and seeds, and be aware of the different effects of apples on diarrhea and constipation. Dairy products: Choose low-lactose or lactose-free products, and pay attention to the risk of dysbacteriosis. Yogurt can help balance the flora. Aquatic products: Avoid raw seafood, consume cooked river fish in moderation during the active period, and consume as appropriate during the remission period. Meat: Intake in moderation, preferably low-fat meat, avoid red meat, and choose stewing as the cooking method. Avoid oily, spicy, cold and raw foods, as well as high-fiber foods such as nuts and beans. Quick-frozen food, fast food, alcohol, coffee, and carbonated drinks should be avoided as much as possible. Tea can be consumed in moderation, and black tea and Pu'er tea are recommended. | For the diet of IBD patients, all foods should be in moderation: 1 Staple food of rice and flour: Gluten protein in flour may cause allergies in some patients, so it is necessary to pay attention to allergic reactions. Eat less during the active phase, and adjust the intake according to the stool condition during the remission phase; 2. Vegetables: may aggravate abdominal pain and diarrhea during the active phase, so it is advisable to eat less. Individuals at risk of stenosis/perforation should refrain from eating to prevent exacerbation of their condition. During the remission period, consume as appropriate, based on the formation of soft stools. For constipation, increase the intake appropriately. 3. Fruits: Avoid raw fruits during active phase, and eat less. For those with high risk of stenosis/perforation due to Crohn's disease, it is advisable to avoid eating fruits; During the remission period, consume in moderation, remove the skin and seeds, chew slowly, or extract the juice; Apple has the effect of astringency and antidiarrheal. It can be eaten more for diarrhea, but should be eaten with caution for constipation; Bananas and mangoes are high in fiber, so they should be eaten less when suffering from diarrhea. Dairy products: Avoid consuming dairy products that have not been pasteurized. 5. Aquatic products: IBD patients are forbidden to eat raw seafood and river fish such as Yusheng during both active and remission periods. Even for cooked seafood, patients with active IBD should eat in moderation. Meat: Red meat from cattle, sheep, and dogs may promote inflammation in IBD, and should be consumed with caution. 7. Greasy and spicy foods: Greasy foods may increase the burden of digestion, and spicy foods may irritate the intestines, both of which should be avoided as much as possible. 8. Cold and raw foods: Cold and raw foods may cause intestinal dysfunction, especially during the active phase of IBD, and should be avoided. 9. Nuts, beans and bean products: Patients with active IBD should not eat nuts, while patients with remission IBD can eat nuts in moderation. 10. Quick-frozen food and fast food: usually contain a large amount of additives and preservatives, which are not good for IBD patients and should be avoided as much as possible. Alcohol and caffeine: Alcohol and caffeine may irritate the intestines and exacerbate IBD symptoms, and should be avoided during both active and remission periods. Carbonated drinks: The sugar and additives in carbonated drinks may be detrimental to IBD patients and are recommended to be avoided. | The diet of IBD patients cannot be generalized, and all foods should follow the principle of moderation. Specific suggestions are as follows: 1. Staple food: IBD patients are not restricted in their intake of rice and pasta, but if you are allergic to gluten protein, you cannot consume pasta. 2. Fruits and vegetables: Patients with active IBD should eat less or not eat fruits and vegetables; In principle, patients with intestinal stenosis and/or penetrating lesions cannot eat vegetables. Patients with IBD in remission can eat fruits and vegetables in moderation, subject to the condition that their stool is soft and can be discharged smoothly. If the stool is loose, it is advisable to reduce the amount of fruits and vegetables. If the stool is hard, it is advisable to increase the amount of fruits and vegetables. 3. Meat: All meat products should be cooked thoroughly before consumption. 4. Dairy products: Due to differences in processing techniques, fat content, and food additives, there are significant differences between different types of dairy products; To avoid the risk of potential infections, IBD patients should avoid consuming unpasteurized dairy products. 4. Aquatic products: IBD patients are forbidden to eat Yusheng and other seafood and river fish. 5. Nuts: Patients with active IBD should not eat nuts, while patients with remission IBD can eat nuts in moderation. 6. Snacks and drinks: usually contain a large amount of food additives and preservatives, which should be avoided as much as possible. |
| 3. How should I determine if the current treatment is working for me? | Evaluating the effectiveness of IBD treatment plans requires a comprehensive consideration of multiple aspects. Including: 1 Observing and recording symptoms is the first step in evaluating treatment effectiveness. The main symptoms include abdominal pain, diarrhea, and bleeding. Effective treatment should significantly reduce or eliminate these symptoms. 2. Improvement in quality of life is an important indicator of successful treatment. Patients should be able to resume normal daily activities and work, reducing the inconvenience caused by the disease. Daily activities: Assess whether it is possible to resume normal daily activities, including housework, sports, and social activities. Effective treatment should make these activities no longer seriously affected by the disease. Work and study: Patients should be able to work or study normally, without frequent leave or rest due to illness. Weight stability or increase is another important indicator. IBD often leads to malnutrition and weight loss, so returning to normal weight is one of the signs of effective treatment. Weight monitoring: Regularly weigh yourself to observe whether there is a stable or rising trend. Weight gain indicates that the absorption of nutrients has improved and the disease is under control. 4. The inflammatory markers in the blood can objectively reflect the level of inflammation in the body. C-reactive protein (CRP) and erythrocyte sedimentation rate (ESR): These are common inflammatory markers. Effective treatment should lead to a decrease in these values, indicating reduced inflammation in the body. 5. Imaging tests, such as colonoscopy, MRI, and CT scan, can visually observe inflammation and lesions in the intestine. Endoscopy: Colonoscopy can directly observe the condition of the intestinal mucosa. When the treatment is effective, the endoscopy should show that the inflammation is reduced or disappeared and the mucosa is healed. Imaging examination: MRI and CT scan can show the thickness of the intestinal wall and the extent of the lesion, assess changes before and after treatment, and determine whether inflammation has been alleviated. By integrating information from all aspects, the effectiveness of the treatment plan can be comprehensively evaluated. | Determining whether an IBD treatment plan is effective requires a comprehensive consideration of multiple aspects, including changes in symptoms, objective examination results, and overall health status. 1. Pay attention to observe whether the symptoms such as abdominal pain, diarrhea, blood in the stool, weight loss, and fatigue have improved or disappeared. Symptom diary: recording daily symptoms, frequency of bowel movements, weight changes, and diet, which helps to track long-term changes in the condition and evaluate treatment effectiveness. 2. Check whether the inflammatory markers such as C-reactive protein (CRP) and erythrocyte sedimentation rate (ESR) have decreased, indicating whether inflammation has been controlled. Fecal examination: fecal calprotectin detection can reflect the degree of intestinal inflammation. If the level decreases, it indicates that the inflammation has been alleviated. Endoscopic examination: Regular colonoscopy and small intestine endoscopy can directly observe the healing of intestinal mucosa. Mucosal healing is an important therapeutic goal, indicating that inflammation has been effectively controlled. 3. Regular follow-up visits allow doctors to evaluate the treatment effect through clinical examination and inquiring about changes in the patient's condition. Scoring systems: Doctors may use some scoring systems, such as the CDAI Crohn's Disease Activity Index or the Mayo score for ulcerative colitis, which are based on symptoms and test results to help assess disease activity. 4. Evaluate whether the activities of daily living have returned to normal, and whether work, study, and social activities have been affected. Emotional and psychological state: Observe whether the emotional state is stable and whether anxiety and depression have been alleviated. 5. Monitor weight and nutritional status to assess whether there are signs of malnutrition. Effective treatment should improve nutrient absorption, maintain stable weight or increase it. Appetite: whether the appetite has improved and the diet is normal. 6. Treatment compliance and side effects: Drug compliance: Ensure that you take the medication on time and in the correct amount. If you interrupt the medication due to side effects or other reasons, it may affect the therapeutic effect. Monitoring of side effects: Assess whether the side effects of the drug are within a controllable range, and whether the reduction or acceptance of side effects is an aspect of effective treatment. By integrating information from multiple aspects, one can more fully determine whether the current treatment plan is effective for them. | IBD patients can judge whether the current treatment plan is effective through various methods and indicators. Including: 1 Symptom relief: Reduced abdominal pain and diarrhea: Pay attention to whether the frequency and intensity of abdominal pain have decreased, and whether the number of diarrhea episodes has decreased. Blood and mucus reduction in stool: Observe the reduction of blood and mucus in stool. Weight change: stable or increasing weight indicates that the absorption of nutrients and intestinal function have improved. Reduction of fatigue and general symptoms: feeling of energy recovery, reduction of fatigue and weakness. 2. Improvement of quality of life: Recovery of daily activities: Able to resume normal work, study, and social activities. Improved sleep quality: sleep becomes more regular and deeper, and there is no longer frequent need to get up at night to use the bathroom. 3. Clinical examination: Endoscopic examination results: Through colonoscopy or other endoscopic examinations, observe the healing of intestinal mucosa, reduce inflammation, ulcers, and bleeding. Imaging examination: such as magnetic resonance imaging (MRI) or CT scan, to evaluate the changes in inflammation and complications inside and outside the intestine. 4. Laboratory indicators: inflammatory markers: the levels of C-reactive protein (CRP) and erythrocyte sedimentation rate (ESR) in the blood decrease, indicating a reduction in inflammation in the body. Fecal calprotectin levels: A decrease in this indicator usually means less inflammation in the gut. Blood tests: to check the levels of hemoglobin, albumin and other nutritional indicators to assess the presence of anemia or malnutrition. 5. Doctor evaluation: Regular follow-up: Regular follow-up with a gastroenterologist, who comprehensively evaluates the therapeutic effect through clinical examination and patient feedback. Questionnaire survey: Use disease activity scoring systems, such as the Harvey-Bradshaw index for Crohn's disease or the Mayo score for ulcerative colitis, to systematically assess the degree of disease activity. 6. Side effects of drugs: monitoring of side effects: pay attention to and record possible side effects of drugs, such as abnormal liver function and increased risk of infection, and communicate with the doctor in a timely manner to adjust the treatment plan. 7. Control of complications: Improvement of extraintestinal manifestations: such as joint pain, skin problems, eye symptoms, and other extraintestinal manifestations, indicate that overall inflammation is well controlled. Through comprehensive evaluation of the above methods, the effectiveness of the current treatment plan can be accurately judged. | IBD patients need to integrate multiple aspects of assessment results to determine whether the current treatment plan is effective. The evaluation can be conducted from the following aspects: 1. Symptom improvement: Observe whether your symptoms have improved, such as abdominal pain, diarrhea, and blood in the stool. If these symptoms are alleviated or disappear, then the treatment plan may be effective. 2. Inflammatory markers: Regular blood tests should be conducted to observe whether the levels of inflammatory markers such as C-reactive protein, fecal calprotectin, and erythrocyte sedimentation rate have decreased. If the level of inflammatory markers decreases, it indicates that inflammation has been controlled and the treatment plan may be effective. 3. Imaging examination: Through imaging examinations such as CT and MRI, observe whether the degree of inflammation, location of lesions, and type of lesions in the intestine have improved. If the intestinal inflammation is reduced or disappears, then the treatment plan may be effective. 4. Endoscopy: For patients with IBD, endoscopy is an important means of evaluating treatment efficacy. Through endoscopy, the degree of inflammation and ulcer healing of the intestinal mucosa can be visually observed. If the inflammation of the intestinal mucosa is alleviated and the ulcer heals, it indicates that the treatment plan is effective. 5. Nutritional status: IBD patients often have problems with malnutrition, so observing whether their nutritional status has improved is also an important aspect of evaluating the effectiveness of treatment plans. If weight gain and improved nutritional status occur, it indicates that the treatment plan may be effective. In addition, patients can also assess the changes in their scores before and after treatment using tools such as the IBD self-assessment questionnaire, such as the SCCAI and HBI, to determine whether the current treatment plan is effective for them. | IBD patients can assess the effectiveness of current treatment through various aspects. This includes observing whether symptoms such as abdominal pain, diarrhea, and blood in the stool have alleviated, whether the results of colonoscopy show reduced inflammation and ulcer healing, whether biochemical indicators such as blood and stool routine tests have improved, and whether there are any side effects from medication. In addition, diet adjustment and improvement of psychological state are also key factors in evaluating the therapeutic effect. By comprehensively evaluating these aspects, patients can better understand the effectiveness of treatment, and if the treatment is not effective, they should seek medical attention early to adjust the treatment plan. | To evaluate the effectiveness of IBD treatment, it is necessary to consider multiple aspects of information, including improvement in clinical symptoms, improvement in laboratory indicators, endoscopic evaluation, quality of life assessment, and safety assessment. Through comprehensive evaluation, the therapeutic effect can be more accurately judged, and personalized treatment plans can be provided for patients. The main clinical symptoms are abdominal pain, diarrhea, and hematochezia. If these symptoms alleviate or disappear, then the treatment plan may be effective. Laboratory indicators mainly include inflammatory markers such as C-reactive protein (CRP) and erythrocyte sedimentation rate (ESR). If the level of inflammatory markers decreases, it indicates that inflammation has been controlled. Endoscopy is an important means of evaluating the therapeutic effect, which can observe the healing of intestinal mucosa, including ulcer healing and inflammation reduction. In addition, it is also important to assess the patient's quality of life through a scale. |
| 4. How often do I need to have a colonoscopy? | The frequency of colonoscopy depends on the type of IBD, the severity and duration of the disease, and the presence of other risk factors. After the initial diagnosis of a newly diagnosed patient, doctors usually recommend a colonoscopy within a certain period of time after the start of treatment to assess the extent and severity of the disease. Patients in stable condition who have good disease control and no severe symptoms are generally recommended to undergo colonoscopy every 1-3 years. This can help monitor intestinal health, prevent complications, and evaluate treatment effectiveness. If symptoms worsen or complications are suspected, more frequent examinations may be needed to assess disease activity and adjust treatment plans. Patients who have been ill for more than 8-10 years, especially those with ulcerative colitis or Crohn's disease involving the entire colon, have an increased risk of colon cancer. Doctors may recommend a colonoscopy every 1-2 years, including sampling and biopsy to monitor precancerous lesions such as intestinal dysplasia. | It is very important for IBD patients to have regular colonoscopy. The frequency may vary depending on the specific situation and medical history: if you have just been diagnosed with IBD, you will usually have an enteroscopy every 1-2 years for the first 8 years after diagnosis; If the condition is stable and there is no family history of colon cancer, an intestinal endoscopy can be performed every 1-2 years. If the disease activity is frequent or severe, more frequent colonoscopy may be needed. For example, once a year. Patients with a long disease history: For patients with a disease history of more than 8-10 years, especially those with pan-colitis or extensive Crohn's disease, it is recommended to undergo colonoscopy every 1-2 years to monitor cancer risk. If there is a family history of colon cancer, more frequent colonoscopies may be needed, usually every 1-2 years. If atypical hyperplasia or polyps were found during previous examinations, it may be necessary to have an examination every 6 months to 1 year. | For patients with IBD, it is important to have regular colonoscopy to monitor the progression of the disease and screen for the risk of colon cancer. The specific frequency of colonoscopy varies depending on individual circumstances and medical history. Patients with stable disease who have no family history of colon cancer are usually advised to undergo colonoscopy every 1-2 years. Patients with unstable disease or a family history of colon cancer: If the disease is active or there is a family history of colon cancer, more frequent colonoscopies may be needed, sometimes once a year. Patients with a long disease history: For patients with a disease history of more than 8-10 years, especially those with pan-colitis or extensive Crohn's disease, it is recommended to undergo colonoscopy every 1-2 years to monitor the risk of cancer. If atypical hyperplasia or polyps were found during previous examinations, more frequent follow-ups may be necessary. | The timing of the colonoscopy review should be determined based on the patient's condition. Typically, after the initial treatment, depending on the treatment plan, which may include 5-aminosalicylic acid, immunosuppressants, and biologics, a colonoscopy review is typically conducted within three to six months to evaluate the effectiveness of the treatment plan. If the review results are good, it is recommended to have a follow-up examination every six months or a year depending on the specific condition of the patient. When there are changes in the condition, such as abdominal pain, diarrhea, or abnormal blood and stool test indicators, it is necessary to come to the hospital for treatment and decide whether an enteroscopy is needed based on the doctor's judgment. In addition, patients with IBD with a disease duration of more than 8 years should undergo tumor screening under colonoscopy; Patients with primary sclerosing cholangitis (PSC) should undergo endoscopic tumor screening while confirming PSC. After the initial colonoscopy screening, the interval between subsequent colonoscopy screenings should be determined based on the risk of disease. The recommended intervals for colonoscopy screening for high, medium, and low-risk patients are annually, every 2-3 years, and every 5 years, respectively. | Patients with ulcerative colitis are advised to undergo endoscopic re-examination during the third and fourth months of the induction phase of drug treatment; Maintain endoscopic reexamination in the early stage of 6-12 months; Endoscopic examination should be conducted during the 12th to 24th months of the long-term stable period. Patients with Crohn's disease who have upper gastrointestinal lesions are advised to undergo gastroscopy after 3-6 months; Patients treated with hormone-immunosuppressant therapy should undergo a full colonoscopy or small intestinal endoscopy after 12 months; Patients treated with biological agents should have endoscopy rechecked after 6-9 months. The efficacy was judged based on the results of the reexamination, and the follow-up endoscopic reexamination time was determined. Given that the treatment of Crohn's disease is a dynamic and relatively long-term process, and there is a certain degree of heterogeneity among IBD patients, the aforementioned endoscopic review time points are recommended for reference. Specific decisions can be made by clinicians based on individual patient conditions. In addition, all IBD patients should undergo colonoscopy screening 8 years after the onset of the first symptoms to assess the extent of the disease and rule out dysplasia. Patients with a family history of colorectal cancer with high-risk characteristics, a first-degree relative ≤50 years old, colonic stenosis or dysplasia, PSC, and extensive colitis with severe active inflammation should schedule their next colonoscopy within one year; Patients with moderate risk factors for extensive colitis with mild to moderate endoscopic and/or histological inflammation, a family history of colorectal cancer in first-degree relatives, and aged >50 years should schedule their next colonoscopy within 2-3 years; Patients without intermediate/high-risk characteristics should be schedule. Patients with ulcerative colitis are advised to undergo endoscopic re-examination during the third and fourth months of the induction phase of drug treatment; Maintain endoscopic reexamination in the early stage of 6-12 months; Endoscopic examination should be conducted during the 12th to 24th months of the long-term stable period. Patients with Crohn's disease who have upper gastrointestinal lesions are advised to undergo gastroscopy after 3-6 months; Patients treated with hormone-immunosuppressant therapy should undergo a full colonoscopy or small intestinal endoscopy after 12 months; Patients treated with biological agents should have endoscopy rechecked after 6-9 months. The efficacy was judged based on the results of the reexamination, and the follow-up endoscopic reexamination time was determined. Given that the treatment of Crohn's disease is a dynamic and relatively long-term process, and there is a certain degree of heterogeneity among IBD patients, the aforementioned endoscopic review time points are recommended for reference. Specific decisions can be made by clinicians based on individual patient conditions. In addition, all IBD patients should undergo colonoscopy screening 8 years after the onset of the first symptoms to assess the extent of the disease and rule out dysplasia. Patients with a family history of colorectal cancer with high-risk characteristics, a first-degree relative ≤50 years old, colonic stenosis or dysplasia, PSC, and extensive colitis with severe active inflammation should schedule their next colonoscopy within one year; Patients with moderate risk factors for extensive colitis with mild to moderate endoscopic and/or histological inflammation, a family history of colorectal cancer in first-degree relatives, and aged >50 years should schedule their next colonoscopy within 2-3 years; Patients without intermediate/high-risk characteristic | Individualized follow-up plans are developed based on the different diseases, such as Crohn's disease, ulcerative colitis, the complexity of the disease, intestinal fistula, abdominal abscess, anal fistula, disease status, active stage, remission stage, postoperative, evaluation of drug efficacy and monitoring of adverse reactions, as well as non-medical factors such as patients' economic, work, and transportation conditions. For patients with active IBD, they should be reexamined every 3 months, while patients who have just recovered should be reexamined every 6-12 months. Patients who have long-term maintenance of remission can be followed up with endoscopy every 6-12 months or longer; For patients with IBD who are newly diagnosed or undergoing annual follow-up, it is recommended to undergo tumor-related screening. For high-risk patients, such as those with severe inflammation, PSC, or a family history of colorectal cancer, it is recommended to undergo colonoscopy monitoring annually for 8-10 years after the onset of symptoms; For patients with moderate risk, it is recommended to undergo colonoscopy every 2-3 years; Patients at low risk should undergo colonoscopy at least every 5 years |
| 5. What is the use of fecal calprotectin testing? | Faecal calprotectin is a protein released by white blood cells during intestinal inflammation, and its detection plays an important role in the diagnosis and monitoring of IBD. Measuring the level of calprotectin in feces can help assess the activity of intestinal inflammation. High levels of calprotectin usually indicate inflammation in the gut, which is useful for distinguishing IBD from other non-inflammatory gastrointestinal diseases such as irritable bowel syndrome. Furthermore, by regularly measuring fecal calprotectin levels, doctors can monitor disease activity and treatment efficacy, adjust treatment plans, and better manage the condition and prevent recurrence. Therefore, fecal calprotectin is a non-invasive and convenient tool for evaluating and managing IBD. | Faecal calprotectin is an important biomarker for assessing the level of inflammatory activity in IBD. It is a protein released by white blood cells, and when inflammation occurs in the intestine, the level of calprotectin will increase significantly. By measuring the concentration of calprotectin in feces, doctors can non-invasively assess the severity of intestinal inflammation. This detection method is simpler and more convenient than invasive methods such as endoscopy, and causes less pain and risk to patients. Faecal calprotectin testing can also help distinguish between IBD and irritable bowel syndrome (IBS), as IBS usually does not cause elevated calprotectin levels. In addition, changes in fecal calprotectin levels can be used to monitor treatment efficacy and disease activity. If the level increases, it may indicate disease recurrence or worsening inflammation, while a decrease may indicate remission. | Faecal calprotectin is an important biomarker used to assess the level of inflammatory activity in IBD. It is released by white blood cells, and when inflammation occurs in the intestine, the level of calprotectin increases significantly. By measuring the concentration of calprotectin in feces, doctors can non-invasively assess the severity of intestinal inflammation. This detection method is simpler and more convenient than endoscopy, and it causes less pain and risk to patients. Faecal calprotectin testing can also help differentiate between IBD and irritable bowel syndrome (IBS) because IBS does not cause an increase in calprotectin levels. In addition, changes in calprotectin levels can be used to monitor treatment efficacy and disease activity. If the level increases, it may indicate disease recurrence or increased inflammation, while a decrease indicates disease remission. Regular testing of calprotectin can help identify problems early, adjust treatment plans in a timely manner, and better manage the condition of IBD patients. | Faecal calprotectin is a sensitive marker of intestinal inflammation, and tracking the level of calprotectin in the stool is very helpful for the treatment of IBD. This is the result of inflammation of the digestive tract caused by Crohn's disease or ulcerative colitis. When inflammation occurs in the mucosal layer of the intestine, the number of white blood cells increases to fight the inflammation. White blood cells contain calprotectin, which releases its contents during inflammatory reactions to protect human cells from inflammation. When the content of calprotectin in feces increases, it means that there is more inflammation in the intestine. By measuring calprotectin in stool, doctors can begin to diagnose IBD in patients or evaluate disease changes in patients who have been diagnosed with IBD, and adjust treatment plans in a timely manner. | Calprotectin in feces can be used for early diagnosis of IBD and is correlated with disease severity. During the treatment process, monitoring fecal calprotectin can evaluate the therapeutic effect, and if there is no improvement, it may be necessary to adjust the treatment plan. During the recovery period of the disease, regular testing of fecal calcium levels is necessary to monitor for recurrence, which is part of the management of intestinal diseases. | Calprotectin is a protein released by neutrophils that plays a role in detecting intestinal inflammation. When neutrophils migrate to the inflammatory area, they release calprotectin, resulting in an increase in fecal calprotectin. The level of fecal calprotectin is closely related to the activity of IBD. When inflammation occurs, calprotectin levels rise; When inflammation is relieved, the level of calprotectin will decrease. Therefore, by regularly detecting fecal calprotectin, the activity of IBD can be monitored, allowing for the assessment of treatment efficacy and the prediction of disease recurrence. The results of calprotectin detection can guide doctors to develop targeted treatment plans. For example, patients with high levels of calprotectin may need more aggressive treatment measures, such as the use of immunosuppressants or biologics. |
| 6. How can I prevent intestinal infections? | To prevent intestinal infections, it is crucial to maintain good personal hygiene, including frequent hand washing, especially before eating and after using the toilet. In addition, ensure the safety of diet and drinking water, and avoid eating undercooked food and drinking untreated water. Choose dining places with good hygiene conditions and try to avoid eating in places with unclear hygiene conditions. Pay attention to food storage and handling, avoid cross contamination, and regularly clean kitchen utensils and surfaces. Vaccination with relevant vaccines, such as influenza vaccine and pneumonia vaccine, can also enhance the body's resistance. Maintain overall health by enhancing the immune system through a balanced diet, moderate exercise, and adequate sleep to help prevent infections. | IBD patients need to take a series of comprehensive measures to prevent intestinal infections, including maintaining good personal hygiene habits, such as frequent hand washing, especially before eating and after using the toilet. In addition, food safety is crucial, and raw and unclean foods should be avoided, ensuring that food is thoroughly cooked and fruits and vegetables are fully cleaned. Pay attention to drinking water, it is best to drink clean bottled water or water that has been disinfected. Avoid using unnecessary antibiotics, as this may disrupt the balance of gut flora and increase the risk of infection. Maintaining a healthy lifestyle, including a balanced diet, moderate exercise, and adequate sleep, can strengthen the immune system and help resist infections. Regular follow-up with doctors, timely monitoring and management of the condition, and ensuring effective control of the condition can also help prevent infection. In addition, vaccinations, such as flu and pneumonia vaccines, can prevent some common infectious diseases. | IBD patients need to take a variety of measures to prevent intestinal infection. Firstly, it is important to maintain good personal hygiene, especially frequent hand washing, especially before eating and after using the toilet. Pay attention to safety in diet, avoid raw and unclean food, ensure that food is thoroughly cooked, fruits and vegetables should be fully cleaned, and drink clean bottled water or water that has been disinfected. Avoid unnecessary use of antibiotics, as they may disrupt the balance of gut microbiota and increase the risk of infection. Maintaining a healthy lifestyle, including a balanced diet, moderate exercise, and adequate sleep, can strengthen the immune system and improve the ability to resist infections. Regular follow-up with doctors, timely monitoring and management of the condition, and ensuring effective control of the condition can reduce the risk of infection. In addition, vaccinations, such as flu and pneumonia vaccines, help prevent some common infectious diseases. By integrating these measures, IBD patients can better prevent intestinal infections and maintain health. | IBD patients can take the following measures to prevent intestinal infection. First of all, it is very important to change living habits, including cultivating good habits of going to bed early and getting up early, avoiding staying up late for a long time, and maintaining the digestive function of the stomach. In addition, it is necessary to stop smoking and limit alcohol intake to reduce the irritation to the intestinal mucosa and reduce the risk of developing intestinal diseases. Secondly, in terms of diet, it is necessary to ensure regular and quantitative meals, avoid overeating, consume more vegetables and fruits, and reduce the intake of foods containing additives and smoked and salted foods. Finally, moderate outdoor sports and exercise can help promote gastrointestinal motility, enhance immunity, and play a positive role in preventing intestinal infections. These lifestyle changes and healthy eating habits can help IBD patients maintain gut health and reduce the risk of infection. | IBD patients need to maintain a healthy lifestyle and pay attention to personal hygiene to prevent intestinal infections. Maintain a regular schedule. Eat a balanced diet and abstain from smoking and drinking. Wash your hands frequently, especially before and after touching food, going to the toilet, and handling animal feces. Avoid eating unclean food, such as raw water, raw meat, and seafood. In addition, according to the doctor's advice, relevant vaccines such as influenza vaccine and pneumonia vaccine should be vaccinated to reduce the aggravation of intestinal inflammation caused by infection. | For IBD patients, preventing intestinal infections is crucial, as it helps control the disease, reduce complications, and improve quality of life. Firstly, food hygiene is crucial. Avoid eating undercooked or unclean food, keep tableware clean and maintain personal hygiene to reduce the risk of bacterial infection. Secondly, avoid stimulating foods such as spicy foods, caffeine, and alcohol, as they may exacerbate intestinal inflammation. High-fiber foods should also be approached with caution, as they may impose unnecessary strain on sensitive digestive systems. Third, moderate exercise can help enhance immunity, promote digestion and absorption of food, and benefit intestinal health. Maintaining a joyful mood also helps reduce intestinal stress reactions. The most important thing is that if there are symptoms of intestinal infection, you should seek medical treatment as soon as possible to avoid the deterioration of the condition. |
| 7. How can I relieve my anxiety and pessimism? | Alleviating anxiety and pessimism can be achieved through various methods. Firstly, maintaining regular exercise can help release stress and improve mood. Secondly, adopting a healthy lifestyle, including a balanced diet and adequate sleep, is very important for mental health. Practicing relaxation techniques such as deep breathing, meditation, or yoga can also help alleviate stress. Maintaining contact with family and friends and seeking their support can provide emotional comfort. In addition, professional counseling or therapy may be particularly effective in dealing with long-term anxiety and pessimism. Appropriate medication is used when necessary to help manage these emotions. Through these comprehensive methods, mental health can be gradually improved, and confidence and coping ability in the face of illness can be enhanced. | There are many ways for IBD patients to alleviate anxiety and pessimism. Firstly, understanding and accepting one's condition is crucial. Actively learning about IBD can help alleviate fear and uncertainty about the disease. Seeking professional psychological support, such as consulting a psychiatrist or participating in a support group, can provide emotional support and practical coping strategies. In addition, it is also important to maintain a healthy lifestyle, regular exercise, adequate sleep and balanced diet to help improve overall mood and physical condition. Practicing relaxation techniques such as deep breathing, meditation, and yoga can effectively alleviate stress and anxiety. Establish a good social network and maintain contact with family, friends, and other IBD patients to share experiences and receive support. At the same time, developing a practical and feasible plan for life and treatment, actively communicating with doctors, and jointly managing the condition can help enhance the sense of control over the future. Finally, try to cultivate hobbies and interests, distract yourself, and enjoy the beautiful things in life. This can help improve your mood and alleviate anxiety and pessimism. | IBD patients can alleviate anxiety and pessimism through various methods. Firstly, understanding and accepting one's condition is an important step. Actively learning about IBD can alleviate uncertainty and fear about the disease. Seeking psychological support, such as consulting a psychologist or participating in a support group, can provide emotional assistance and practical coping strategies. Maintaining a healthy lifestyle can also help improve mood. Regular exercise, adequate sleep, and a balanced diet can enhance the body's resistance and emotional stability. Practicing relaxation techniques such as deep breathing, meditation, and yoga can effectively alleviate stress and anxiety. Staying connected with family, friends and other IBD patients to share experiences and receive support can boost confidence and coping skills. Developing a practical living and treatment plan, actively communicating with doctors, and jointly managing the condition can enhance the sense of control over the future. Finally, cultivating hobbies and participating in enjoyable activities can divert attention, enhance mood, and make life more rich and fulfilling. Through these methods, IBD patients can better cope with anxiety and pessimism and improve their quality of life. | First of all, patients should have a correct and sufficient understanding of IBD, including the nature of the disease, treatment methods, and prognosis, to avoid excessive fear and pessimism about the disease. Secondly, patients need to establish a positive attitude and seek psychological support when necessary. For example, joining an IBD patient support group or community, and seeking professional psychological help, can help cope with the psychological stress caused by chronic diseases. Third, maintain a regular schedule and moderate exercise, and learn and practice relaxation techniques such as meditation, deep breathing exercises, and yoga. Through these comprehensive methods, we can gradually improve mental health and enhance our confidence and ability to cope with diseases. | Patients with IBD are often plagued by depression and pessimistic emotions, which can negatively affect treatment and recovery. To alleviate these emotions, patients can take a series of measures. First, seek professional psychological counseling and treatment to address emotional issues such as anxiety and depression, and enhance one's self-coping skills. Secondly, share with friends and relatives to alleviate loneliness and stress and obtain emotional support. Regular participation in social activities such as IBD patient support groups can help expand social circles and improve quality of life. Third, moderate exercise, such as jogging, walking, yoga or tai chi, can promote physical and emotional relaxation. Lastly, traditional Chinese medicine treatments such as acupuncture and moxibustion, massage, and herbal medicine can help patients balance their physical state and alleviate depression and pessimism. | To alleviate anxiety and pessimism in IBD patients, it is necessary to establish a correct understanding of the disease, maintain an optimistic and positive attitude towards life, maintain communication with family and friends, and obtain help and care from others. At the same time, you can seek professional psychological help, such as cognitive behavioral therapy (CBT) or other psychological treatment methods, to help cope with the psychological stress caused by chronic diseases. Third, learn relaxation techniques such as meditation and yoga to improve your mental health and face the challenges of the disease positively. |
| 8. Will I need lifelong treatment? | Whether lifelong treatment is needed depends on the severity of IBD and individual response. For many patients, IBD is a long-term chronic condition that requires ongoing management and treatment to control symptoms, prevent relapse, and manage complications. While some patients may reduce their medication use during remission, stopping completely usually leads to relapse. Therefore, regular follow-up and long-term treatment as prescribed by a doctor are necessary for most IBD patients. | IBD patients usually need lifelong treatment. Although there is currently no complete cure for IBD, appropriate treatment can effectively control symptoms, reduce inflammation, and prevent complications. The goal of treatment is to achieve and maintain remission, reduce symptoms, improve quality of life, and prevent relapse as much as possible. Treatment options may include medication, dietary adjustments, lifestyle changes, and sometimes surgery. Because the disease may fluctuate, it is very important to follow up with the doctor regularly, monitor the condition and adjust the treatment plan. Despite the need for long-term treatment, most people with IBD are able to lead relatively normal and fulfilling lives through active management. | IBD patients usually need lifelong treatment. IBD cannot be completely cured at present, but through continuous treatment, symptoms can be effectively controlled, inflammation can be reduced, and complications can be prevented. The goal of treatment is to achieve and maintain remission, improve quality of life, and reduce the frequency of disease recurrence. Treatment may include medications, dietary adjustments, and lifestyle changes, and sometimes surgical intervention may be necessary. Because the disease may fluctuate over time, it is important to regularly follow up with the doctor, monitor the condition, and adjust the treatment plan as needed. Despite the need for long-term treatment, many people with IBD are able to lead relatively normal and fulfilling lives with active management of their condition. | Whether IBD patients need lifelong treatment depends on the specific circumstances of the patient. This is because IBD is a chronic, recurring disease that requires long-term monitoring and management to control inflammation, alleviate symptoms, and prevent complications. However, the specific treatment plan and duration should be determined based on the patient's specific condition and the doctor's advice. | Generally, IBD patients need long-term medication and regular follow-up, but whether it is for life or for several years depends on the type of disease and the severity of the disease. Crohn's disease and ulcerative colitis are chronic and recurrent diseases that are currently incurable. If the medication is discontinued or the dosage does not meet the treatment standard, the disease may flare up suddenly. | We must acknowledge that IBD is a disease that cannot be fully cured. Although active treatment can alleviate symptoms and reduce the occurrence of complications, patients still need to manage the disease for a long time and maintain good habits and mentality. With the deepening of medical research, new treatment methods and means are constantly emerging, which will bring more hope and choices to patients. |
| 9. What can I do to prevent a relapse? | Preventing the recurrence of IBD requires the comprehensive consideration of drug therapy, lifestyle and dietary adjustments, as well as regular medical follow-ups. First of all, you should take the medicine on time according to the doctor's advice, even if you feel good, and do not stop the medicine by yourself. You should also have regular follow-ups to adjust the treatment plan. In terms of diet, choose foods that are easy to digest and have balanced nutrition, avoid known trigger foods, take small meals to reduce the burden on the intestines, and supplement vitamins and minerals when needed. At the same time, quitting smoking, moderate exercise and ensuring adequate sleep can help strengthen the body and promote intestinal health. Use yoga, meditation and other methods to manage stress, and seek psychological support to alleviate the psychological stress and anxiety caused by long-term illnesses. To avoid infection, maintain good hygiene habits, receive regular vaccinations, and seek medical treatment for infection symptoms in a timely manner. Regular blood tests, stool tests, imaging tests, and colonoscopy are performed to monitor disease activity. Understanding the basic knowledge of IBD, enhancing self-management skills, working closely with doctors, nurses, and dietitians to develop and adjust personalized treatment and management plans are key to preventing recurrence. | A series of comprehensive measures are needed to prevent recurrence in IBD patients. Firstly, it is important to follow the doctor's treatment plan, including taking medication on time and regular follow-ups. Drug therapy usually includes anti-inflammatory drugs, immunosuppressants, and biologics, which help control inflammation and prevent recurrence. Regular monitoring of the disease condition, through blood tests, stool tests, and imaging tests, can help detect disease activity early and adjust treatment plans in a timely manner. In terms of diet, it is important to maintain a balanced diet and avoid foods that may trigger symptoms. For example, certain high-fat foods, dairy products, spicy foods, and high-fiber foods may worsen symptoms. A food diary can help patients identify and avoid these triggers. Lifestyle changes are also key to preventing recurrence. Maintaining a regular schedule, appropriate exercise, and adequate rest can help strengthen the immune system and overall health. Avoid smoking and excessive alcohol consumption, as these habits can exacerbate intestinal inflammation and increase the risk of recurrence. Managing stress is also crucial for IBD patients. You can reduce stress by practicing yoga, meditation, deep breathing, and other relaxation techniques. Psychological support and counseling can help patients cope with the emotional stress and anxiety caused by the disease. Finally, establish good communication with your doctor and report any new symptoms or changes in a timely manner to ensure that a series of comprehensive measures are needed to prevent recurrence in IBD patients. Firstly, it is important to follow the doctor's treatment plan, including taking medication on time and regular follow-ups. Drug therapy usually includes anti-inflammatory drugs, immunosuppressants, and biologics, which help control inflammation and prevent recurrence. Regular monitoring of the disease condition, through blood tests, stool tests, and imaging tests, can help detect disease activity early and adjust treatment plans in a timely manner. In terms of diet, it is important to maintain a balanced diet and avoid foods that may trigger symptoms. For example, certain high-fat foods, dairy products, spicy foods, and high-fiber foods may worsen symptoms. A food diary can help patients identify and avoid these triggers. Lifestyle changes are also key to preventing recurrence. Maintaining a regular schedule, appropriate exercise, and adequate rest can help strengthen the immune system and overall health. Avoid smoking and excessive alcohol consumption, as these habits can exacerbate intestinal inflammation and increase the risk of recurrence. Managing stress is also crucial for IBD patients. You can reduce stress by practicing yoga, meditation, deep breathing, and other relaxation techniques. Psychological support and counseling can help patients cope with the emotional stress and anxiety caused by the disease. Finally, establish good communication with your doctor and report any new symptoms or changes in a timely manner | Comprehensive measures are needed to prevent recurrence in IBD patients. First of all, strictly following the doctor's treatment plan is the key. Taking medication on time, regular follow-ups, and timely adjustment of treatment plans can effectively control inflammation and reduce the risk of recurrence. In terms of diet, it is important to maintain a balanced diet. It is recommended to avoid foods that may trigger symptoms, such as high-fat, high-sugar, spicy, dairy, and high-fiber foods. Everyone responds differently to food, and you can discover and avoid trigger foods by keeping a food diary. Eating more easily digestible and nutritious foods can help maintain intestinal health. The adjustment of lifestyle cannot be ignored. Maintaining a regular schedule and appropriate exercise can help strengthen the immune system and overall health. Adequate sleep and moderate exercise can reduce stress and enhance physical strength. Avoid smoking and excessive alcohol consumption, as these habits can exacerbate intestinal inflammation and increase the likelihood of recurrence. Managing stress is especially important for people with IBD. Long-term psychological stress and anxiety may induce or exacerbate symptoms. Relaxation techniques such as yoga, meditation, and deep breathing can effectively reduce stress. Seeking psychological support and participating in support groups can help cope with emotional distress caused by the disease. Maintain good communication and comprehensive measures are needed to prevent recurrence in IBD patients. First of all, strictly following the doctor's treatment plan is the key. Taking medication on time, regular follow-ups, and timely adjustment of treatment plans can effectively control inflammation and reduce the risk of recurrence. In terms of diet, it is important to maintain a balanced diet. It is recommended to avoid foods that may trigger symptoms, such as high-fat, high-sugar, spicy, dairy, and high-fiber foods. Everyone responds differently to food, and you can discover and avoid trigger foods by keeping a food diary. Eating more easily digestible and nutritious foods can help maintain intestinal health. The adjustment of lifestyle cannot be ignored. Maintaining a regular schedule and appropriate exercise can help strengthen the immune system and overall health. Adequate sleep and moderate exercise can reduce stress and enhance physical strength. Avoid smoking and excessive alcohol consumption, as these habits can exacerbate intestinal inflammation and increase the likelihood of recurrence. Managing stress is especially important for people with IBD. Long-term psychological stress and anxiety may induce or exacerbate symptoms. Relaxation techniques such as yoga, meditation, and deep breathing can effectively reduce stress. Seeking psychological support and participating in support groups can help cope with emotional distress caused by the disease. | First of all, IBD patients and their families should establish the concept that IBD is a chronic disease that should be treated persistently, which is crucial for preventing the recurrence of IBD. Secondly, it is necessary to actively communicate with the doctor and follow the treatment plan given by the doctor. Maintain a regular sleep schedule, engage in moderate exercise, and get enough rest to avoid staying up late and overexerting oneself. Pay attention to maintaining proper nutrition, eating regularly, and avoiding foods that are not conducive to recovery or unclean. After remission through formal clinical treatment, it is not advisable to take it lightly, as the clinical characteristics of the disease are recurrent attacks. During the remission period, it is still necessary to strictly follow the doctor's advice to take medication regularly and in a quantitative manner, and not to increase or decrease the dosage or stop taking medication without authorization. Even if there are no symptoms, IBD patients need regular follow-ups to assess the disease status and adjust the treatment plan in a timely manner. | In order to effectively prevent the recurrence of IBD, patients need to first establish a positive attitude towards life, quit smoking and limit alcohol intake, and maintain healthy lifestyle habits. In terms of diet, you should choose balanced, light, and easily digestible foods, avoid eating stimulating and difficult-to-digest foods, and pay attention to supplementing sufficient nutrients. Maintaining an optimistic attitude is also important for preventing the recurrence of IBD. You can reduce psychological stress by participating in social activities and undergoing psychological counseling. In addition, patients should strictly follow the doctor's treatment recommendations, take medication on time, and not stop taking medication or change the treatment plan at will. Regular follow-up visits and necessary examinations are key measures to prevent the recurrence of IBD. They can detect changes in the condition in a timely manner, adjust treatment plans, and reduce the risk of recurrence. At the same time, patients should also pay attention to strengthening their physical fitness, engaging in appropriate physical exercise, and improving their body immunity. Avoiding overwork and abdominal coldness is also an important measure to prevent the recurrence of IBD. By integrating the above measures, patients can effectively reduce the risk of IBD recurrence, improve their quality of life, and enjoy a healthy life. | Preventing the recurrence of IBD requires the comprehensive consideration of drug therapy, lifestyle and dietary adjustments, as well as regular medical follow-ups. Firstly, patients need to strictly follow the treatment plan and not stop taking medication or change the dosage on their own. Secondly, it is important to maintain good living habits, including quitting smoking, avoiding overwork, and maintaining a positive and optimistic attitude. Pay special attention to diet, choose balanced and easily digestible foods, and avoid foods that aggravate intestinal inflammation. Patients should also strengthen physical exercise, enhance physical fitness, and improve resistance to better cope with the challenges of IBD. Third, regular follow-up visits are crucial in preventing the recurrence of IBD. Through review, we can timely understand the changes in the patient's condition and adjust the treatment plan. Laboratory tests, imaging tests, and colonoscopy may be necessary for follow-up to monitor disease activity. In short, patients need to adopt scientific self-management and actively communicate with doctors and nurses to help reduce disease recurrence. |
| 10. How can I tell if I am in the acute or remission phase? | It is very important to determine whether you are in the active phase or remission phase of the disease, as this will affect the patient's treatment plan and lifestyle adjustments. The evaluation can be conducted from the following aspects: 1 Symptom evaluation: symptoms in the active phase of the disease: pain, diarrhea, blood in the stool, abdominal bloating, anemia, loss of appetite, etc. Symptoms in remission: Symptoms significantly reduce or disappear, with increased energy, good appetite, and stable weight; 2. Physical examination: inflammatory signs: abdominal tenderness, increased bowel sounds, abdominal tenderness, etc. Nutritional status: weight change, anemia, skin condition, etc. 3. Laboratory examination: Detect inflammatory markers such as C-reactive protein (CRP) and erythrocyte sedimentation rate (ESR), and measure the level of calprotectin in the stool. A high level may indicate intestinal inflammation activity. 4. Colonoscopy directly observes the degree of inflammation and activity inside the intestines. 5. Imaging examination: such as ultrasound, CT scan or MRI, to assess the intestinal structure and inflammation level. 6. Disease activity assessment tool: Crohn's Disease Activity Index, used to assess the activity level of Crohn's disease; The Ulcerative Colitis Activity Index is used to assess the activity level of ulcerative colitis. Of course, the safest way is to communicate with your doctor, describe your symptoms and signs, and receive a comprehensive clinical evaluation. | IBD patients can judge whether they are in the active or remission stage of the disease by evaluating the following aspects. First, pay attention to the changes in symptoms. The active phase of the disease is usually accompanied by significant symptoms, such as frequent diarrhea with blood, abdominal pain, weight loss, fatigue, and loss of appetite. If these symptoms increase significantly, it may mean that the disease is active. Secondly, conduct regular medical check-ups. Doctors will assess the extent of inflammation through blood tests, fecal calprotectin tests, and imaging tests such as endoscopy and MRI. Blood tests can show the level of inflammatory markers, such as C-reactive protein (CRP) and erythrocyte sedimentation rate (ESR), which are usually elevated in patients with increased inflammation. Fecal calprotectin detection is a non-invasive detection method that can reflect the degree of intestinal inflammation. If the level of calprotectin rises, it may indicate increased intestinal inflammatory activity. Again, maintain close communication with the doctor. Regularly visit the doctor and report any changes in symptoms or new symptoms. The doctor will determine whether the condition is in an active or remission stage based on the patient's report and examination results. Additionally, using disease activity scoring tools such as the Simplified Disease Activity Index (SDAI) or Harvey-Bradshaw Index (HBI) can help patients and doctors more systematically assess disease activity. These tools typically include symptom scores and laboratory test results, which can provide an objective reference. Finally, self-observation and recording by the patient is also an important part. Keeping a health log that records daily symptoms, diet, medication use, and other relevant factors can help patients and doctors better understand changes in the condition and potential triggers. | IBD patients can judge whether they are in the active or remission stage of the disease through the following aspects. First, pay attention to the changes in symptoms. During the active phase of the disease, patients often experience significantly worsening symptoms, such as frequent diarrhea that may be bloody or mucous, abdominal pain, weight loss, fatigue, and loss of appetite. If these symptoms increase significantly, it may indicate that the disease is in an active phase. On the contrary, during the remission period, symptoms will decrease or disappear, and patients will feel more normal. Secondly, regular medical check-ups are an important means of judging the condition of the disease. Doctors will assess the degree of inflammation through blood tests, fecal calprotectin tests, and imaging tests such as endoscopy and MRI. Blood tests can show the level of inflammatory markers, such as C-reactive protein (CRP) and erythrocyte sedimentation rate (ESR), which are usually elevated in patients with severe inflammation. The fecal calprotectin test is a non-invasive method that can indicate the extent of intestinal inflammation. If the level of calprotectin increases, it may indicate increased intestinal inflammation activity. It is important to maintain close communication with your doctor. Regular follow-up with the doctor, reporting any changes in symptoms or new symptoms, and the doctor will determine whether the condition is in an active or remission phase based on the patient's report and examination results. Using disease activity scoring tools, such as the Simplified Disease Activity Index (SDAI) or the Harvey-Bradshaw Index (HBI), can help patients and doctors more systematically assess the condition of the disease. These tools typically include symptom scores and laboratory test results, which can provide an objective reference. Finally, self-observation and recording by the patient is also an important part. Keeping a health log that records daily symptoms, diet, medication use, and other relevant factors can help patients and doctors better understand changes in the condition and potential triggers. | Ulcerative colitis can be divided into active and remission stages based on the severity of the disease. From the perspective of clinical symptoms and signs, patients in the active stage often experience vomiting, dehydration, diarrhea, tenesmus, loss of appetite, mucous purulent bloody stool, and abdominal toothache; The diarrhea in remission period may disappear, the abdominal pain is not obvious, there are often 3-4 times of stool with a rough texture, and there are symptoms such as malnutrition, anemia, and weight loss. Secondly, inflammatory markers such as CRP, ESR, and fecal calprotectin can be detected, and high levels may indicate an active phase. Thirdly, the degree of intestinal inflammation can also be assessed through colonoscopy and imaging tests. | The judgment of IBD active and remission stages is mainly based on the patient's clinical manifestations, hematological examination, and the results of colonoscopy. During the active phase, patients often experience typical symptoms of IBD, such as abdominal pain, frequent diarrhea, stool with mucus and pus, and obvious tenesmus. Laboratory tests will reveal elevated levels of inflammatory markers such as CRP, ESR, and calprotectin. During remission, symptoms usually decrease or completely disappear, and inflammatory marker levels return to normal. In addition, imaging tests such as MRI, CT, ultrasound, and endoscopy can also distinguish between active and remission stages. However, it should be emphasized that the specific diagnosis of the condition still requires the doctor to comprehensively consider the patient's medical history, clinical manifestations, and relevant examination results. | The judgment of the active and remission stages of IBD is mainly based on the patient's clinical manifestations, hematological examination, and the results of colonoscopy. During the active phase, patients usually present with obvious clinical symptoms such as abdominal pain, diarrhea, mucus, pus, and bloody stools. Hematological examination indicators may be elevated, and colonoscopy may reveal diffuse inflammatory reactions and ulcers in the mucosa. During the remission phase, the patient's clinical symptoms significantly improve or disappear, hematological examination indicators return to normal, and colonoscopy reveals gradual improvement and repair of colonic inflammation. It should be noted that specific judgment should be made by professional doctors according to the specific situation of the patient. |
